# Identification and validation of immune and diagnostic biomarkers for interstitial cystitis/painful bladder syndrome by integrating bioinformatics and machine-learning

**DOI:** 10.3389/fimmu.2025.1511529

**Published:** 2025-01-23

**Authors:** Tao Zhou, Can Zhu, Wei Zhang, Qiongfang Wu, Mingqiang Deng, Zhiwei Jiang, Longfei Peng, Hao Geng, Zhouting Tuo, Ci Zou

**Affiliations:** ^1^ Department of Urology, The Second Affiliated Hospital of Anhui Medical University, Hefei, China; ^2^ Center for Cell Lineage and Development, Guangzhou Institutes of Biomedicine and Health, Chinese Academy of Sciences Guangzhou, Guangzhou, China; ^3^ Department of Urological Surgery, Daping Hospital, Army Medical Center of PLA, Army Medical University, Chongqing, China

**Keywords:** IC/BPS, bioinformatics, machine-learning, PLAC8, immune cell landscape

## Abstract

**Background:**

The etiology of interstitial cystitis/painful bladder syndrome (IC/BPS) remains elusive, presenting significant challenges in both diagnosis and treatment. To address these challenges, we employed a comprehensive approach aimed at identifying diagnostic biomarkers that could facilitate the assessment of immune status in individuals with IC/BPS.

**Methods:**

Transcriptome data from IC/BPS patients were sourced from the Gene Expression Omnibus (GEO) database. We identified differentially expressed genes (DEGs) crucial for gene set enrichment analysis. Key genes within the module were revealed using weighted gene co-expression network analysis (WGCNA). Hub genes in IC/BPS patients were identified through the application of three distinct machine-learning algorithms. Additionally, the inflammatory status and immune landscape of IC/BPS patients were evaluated using the ssGSEA algorithm. The expression and biological functions of key genes in IC/BPS were further validated through *in vitro* experiments.

**Results:**

A total of 87 DEGs were identified, comprising 43 up-regulated and 44 down-regulated genes. The integration of predictions from the three machine-learning algorithms highlighted three pivotal genes: PLAC8 (AUC: 0.887), S100A8 (AUC: 0.818), and PPBP (AUC: 0.871). Analysis of IC/BPS tissue samples confirmed elevated PLAC8 expression and the presence of immune cell markers in the validation cohorts. Moreover, PLAC8 overexpression was found to promote the proliferation of urothelial cells without affecting their migratory ability by inhibiting the Akt/mTOR/PI3K signaling pathway.

**Conclusions:**

Our study identifies potential diagnostic candidate genes and reveals the complex immune landscape associated with IC/BPS. Among them, PLAC8 is a promising diagnostic biomarker that modulates the immune response in patients with IC/BPS, which provides new insights into the future diagnosis of IC/BPS.

## 1 Introduction

Interstitial cystitis/painful bladder syndrome (IC/BPS) encompasses a spectrum of disorders primarily distinguished by bladder pain and discomfort, accompanied by lower urinary tract symptoms (1). Currently, IC/BPS is classified into two primary types: those with Hunner’s lesions (HIC) and those without (NHIC) (2). Researchers have identified distinct clinical symptoms and diverse treatment responses between these subtypes, indicating potential variations in pathologic mechanisms. While IC/BPS is not fatal, it imposes a considerable burden on affected individuals, particularly women. The etiology of IC/BPS remains elusive, with mounting evidence suggesting a multifaceted pathogenesis involving genetic predisposition, hormonal factors, history of urinary tract infections, autoimmune conditions, and other comorbidities (3–5). The absence of sensitive and specific biomarkers complicates the diagnosis of IC/BPS, underscoring the imperative for reliable markers to enhance accuracy and differentiate IC/BPS from similar diseases.

Clinical pathology strongly supports the classification of IC/BPS as a chronic inflammatory disease that progressively affects the entire bladder wall. The initial stage is primarily characterized by pain symptoms, while advanced stages may involve a significant reduction in bladder capacity (2). Increasing evidence highlights the critical roles of chronic inflammation and immune dysfunction in the progression of IC/BPS (6). Recent studies, utilizing single-cell sequencing analysis, have emphasized the involvement of the inflammatory immune microenvironment and immune cell crosstalk in the pathogenic mechanisms of IC/BPS (7, 8). Neutrophils are key players in early immune responses, typically involved in bacterial infections and acute inflammation. In the bladder tissue of IC/BPS patients, neutrophil infiltration is often elevated, exacerbating the local inflammatory response by releasing various cytokines and chemokines (9). Overactivation of Th1 cells may further drive inflammatory responses, particularly in areas with Hunner’s lesions (10). Additionally, Moldwin et al. observed abnormal immune cell infiltration in IC/BPS tissue samples, alongside elevated levels of pro-inflammatory cytokines in urine samples from these patients (11). For instance, the expression of the chemokine CXCL-10 is significantly elevated in the bladder tissue of IC/BPS patients compared to that of healthy individuals. Despite previous bioinformatics analyses identifying potential molecular markers in IC/BPS patients, a thorough exploration and validation of diagnostic markers and immunological effects are warranted (12–14). Further comprehensive analysis is necessary to unveil differences in the immune landscape between IC/BPS patients and healthy populations.

In this study, we integrated bioinformatics analysis and machine-learning algorithms to identify diagnostic biomarkers contributing to assessing the immune status in IC/BPS patients. Additionally, we validated the expression of key genes and unveiled their relevance to the immune landscape in IC/BPS tissues.

## 2 Methods

### 2.1 Data collection

Transcriptome data for IC/BPS patients were obtained from the Gene Expression Omnibus (GEO) database, encompassing GSE11783 (15), GSE28242 (16), and GSE57560 (17). Key information about these datasets is summarized in **Table 1**. Due to differences in sequencing platforms, methodologies, and experimental designs across the datasets, we applied the “Combat” function to adjust for batch effects arising from these variations. Before applying “Combat,” we first preprocessed the data by removing outliers. The method was then used to correct for batch effects, ensuring that subsequent analyses more accurately reflected biological variations rather than experimental design differences. Following established protocols, the datasets were integrated using the “sva” package in R software, effectively eliminating batch effects for subsequent analysis (18, 19).

**Table 1:** Basic information of the GEO datasets in IC/BPS patients.

GEO accession	Type	Platform	Normal control	IC/BPS
GSE11783	mRNA	GPL570	6	10
GSE28242	mRNA	GPL6244	5	8
GSE57560	mRNA	GPL16699	3	13

IC/BPS, interstitial cystitis/painful bladder syndrome; GEO, Gene Expression Omnibus.

### 2.2 Identification of differentially expressed genes

DEGs between IC/BPS samples and normal samples were identified using the “limma” package in the combined dataset (20). A threshold of P-value < 0.05 and |log2FC| > 1 was applied to screen for DEGs. Adjusted P-value < 0.05 and FDR (Q-value) < 0.25 were set as the cut-off criteria. The expression patterns of these DEGs in both patient groups were then visualized using the “pheatmap” package.

### 2.3 Functional enrichment analysis

To further investigate the biological role of these DEGs, we performed functional enrichment analyses of “clusterProfiler” packages (*P*-value < 0.05), including the Gene Ontology (GO) and Kyoto Encyclopedia of Genes and genes Genomes (KEGG) (21). Additionally, Gene Set Enrichment Analysis (GSEA) was undertaken in both patient groups, utilizing the “clusterProfiler” and “enrichplot” packages. Detailed descriptions of these methods can be found in previous studies (22, 23).

### 2.4 Weighted gene co-expression network analysis

To further analyze potential pathogenic genes in IC/BPS patients, we used the “WGCNA” and “limma” R packages to construct gene co-expression networks and analyze correlations between gene modules and specific phenotypes (20, 24). The key gene of the relevant module is defined as the first principal component gene of each module and is adopted as a representative of all genes in each module (Module membership > 0.80 and *P* < 0.05).

### 2.5 Construction and verification of the diagnostic prediction model

To pinpoint the key pathogenic genes, we employed three machine-learning algorithms—random forest (RF) (25), loss absolute shrinkage and selection operator (LASSO) (26) and support vector machine -recursive feature elimination (SVM-RFE) (27). The analyses for SVM-RFE, RF, and LASSO regression were conducted using the R packages “glmnet,” “kernlab,” and “randomForest” (28–30). Genes situated in the overlapping regions of the three algorithms were deemed crucial in IC/BPS diagnosis. Subsequently, a diagnostic nomogram model was constructed based on these identified genes. To further evaluate the predictive model’s capability in distinguishing between normal and IC/BPS tissue, we conducted decision curve analysis (DCA), calibration curve, and clinical impact curve (CIC) using the R packages “pROC” and “rmda” (31, 32). Additionally, we assessed the studies based on receiver operating characteristic (ROC) curves, calculating the area under the curve (AUC) to measure the predictive power of the key genes. Detailed methodologies are referenced in several prior studies (33).

### 2.6 Immune cells infiltration analysis

Immune cells play a crucial role in the crosstalk that influences disease progression and treatment response. To assess each immune cell across all samples, we utilized the ssGSEA algorithm, incorporating the “GSVA,” “GSEABase,” and “limma” packages. This analysis was based on 28 immune cell gene sets, allowing us to scrutinize and compare the differences between normal and IC/BPS tissues (34).

### 2.7 Collection of clinical samples

Between January 2022 and August 2023, we collected 8 bladder tissue samples from individuals diagnosed with interstitial cystitis/bladder pain syndrome (IC/BPS) and 10 bladder tissue samples from unaffected controls (UC), following previously established methods (11, 35). Cystoscopy was performed on all IC/BPS patients, and biopsies were taken from tissue suspected to contain Hunner’s lesions. UC samples were obtained from patients with benign bladder tumors, with biopsies confirming the absence of inflammatory changes. Laboratory test results, including hemoglobin (Hb), white blood cell (WBC) count, neutrophil-to-lymphocyte ratio (NLR), lymphocyte-to-monocyte ratio (LMR), and absolute lymphocyte count (ALC), were collected within 12 hours prior to biopsy. Additionally, clinical data on age, sex, history of urinary surgery, diabetes, hypertension, smoking, and alcohol consumption were recorded. The classification of samples into IC/BPS or UC groups was independently verified by two pathologists.

### 2.8 Immunohistochemistry assay

The specific immunohistochemical staining methods employed in this study were referenced from our previous studies (22, 36). All tissues were transformed into pathological sections and then processed using an IHC kit as per the manufacturer’s instructions (cat. SP-9001; ZSGB-BIO, China). The primary antibodies utilized in this study included PLAC8 (cat. 12284-1-AP, Proteintech, China), CXCL10 (cat. DF6417, Affinity, USA), c-Kit (CD117) (cat. AF6153, Affinity, USA), SDC1 (CD138) (cat. DF6367, Affinity, USA), CD163 (cat. DF8235, Affinity, USA), CD20 (cat. DF13319, Affinity, USA), CD14 (cat. DF13278, Affinity, USA), FucT-IV (CD15) (cat. DF8547, Affinity, USA), and CD3D (cat. DF6370, Affinity, USA). Two senior pathologists independently evaluated the IHC staining results. The percentage of positive cells was categorized as follows: 1 = 0-25%, 2 = 26%-50%, 3 = 51%-75%, and 4 = 76%-100%. Staining intensity was defined as 0 = no staining; 1 = weak staining; 2 = medium staining; 3 = strong staining. The final IHC score was derived by multiplying the staining percentage and staining intensity. Immune cell staining was assessed based on the number of positive cells within the field of vision.

### 2.9 Cell culture and transfection

Normal human urothelial cell line (SV-HUC-1) was purchased from Procell Life Science & Technology Co., Ltd. (China) using Ham’s F-12 (F-12K) medium modified by Kaighn (Gibco, USA) for cultivation. SV-HUC-1 cell lines were treated with lipopolysaccharide (LPS, 10ug/L, MedChemExpress) and adenosine triphosphate (ATP, 2.5 mM, MedChemExpress) to induce inflammatory damage. We overexpressed PLAC8 using PB-EF1a-FLAG-PLAC8-IRES-Hygromycin vector, and selected PB-EF1a-3FLAG-GFP-IRES-Hygromycin vector as a negative control group. These vectors were purchased from Sigma-Aldrich according to the manufacturer’s instructions.

### 2.10 Western blot analysis

Total proteins were extracted from cells using RIPA lysis buffers of protease and phosphatase inhibitors (Beyotime Biotechnology, China). Pierce BCA protein detection kit was used to determine the protein concentration (Beyotime Biotechnology, China). Protein samples were isolated by sodium dodecyl sulfate polyacrylamide gel electrophoresis and transferred to PVDF membranes (Beyotime Biotechnology, China). Incubate overnight with primary antibodies, including PLAC8 (1:1000; Proteintech, China), Flag (1:1000; Sigma-Aldrich, USA), α-tubulin (1:10000, Sigma-Aldrich, USA), AKT (1:1000; Proteintech, China), p-AKT (1:1000; Proteintech, China), mTOR (1:1000; Proteintech, China), p-mTOR (1:1000; Proteintech, China), and PI3K (1:1000; Proteintech, China). The PVDF membrane was then incubated with the horseradish peroxide-coupled secondary antibody (Beyotime Biotechnology, China). Protein bands were detected using an enhanced chemiluminescence kit (Thermo Fisher, USA).

### 2.11 Cell proliferation assays

Cell proliferation ability was detected using cell counting kit-8 (Beyotime Biotechnology, China). 5000 cells were mixed and inoculated into a 96-well plate incubator for 24h. The absorbance value of 450 nm was measured according to the manufacturer’s instructions.

### 2.12 Wound healing assays

SV-HUC-1 cells were inoculated into the 6-well plate, and subsequent experiments were carried out when the cell confluence reached 100%. Wounds were formed with a 200 μL pipette suction and cell migration was assessed with photographs taken every 12 hours.

### 2.13 Statistical analysis

The statistical analysis was conducted using R software (version 4.2.1), and data processing followed established literature protocols. The unpaired *Student’s t test* was used for statistical comparison between the two groups. The *Kruskal-Wallis test* is used for statistical comparison of non-normally distributed data. A significance level of *P-value* < 0.05 was considered statistically significant.

## 3 Results

### 3.1 Identification of DEGs in IC/BPS patients and functional enrichment analysis

**Figure 1:**
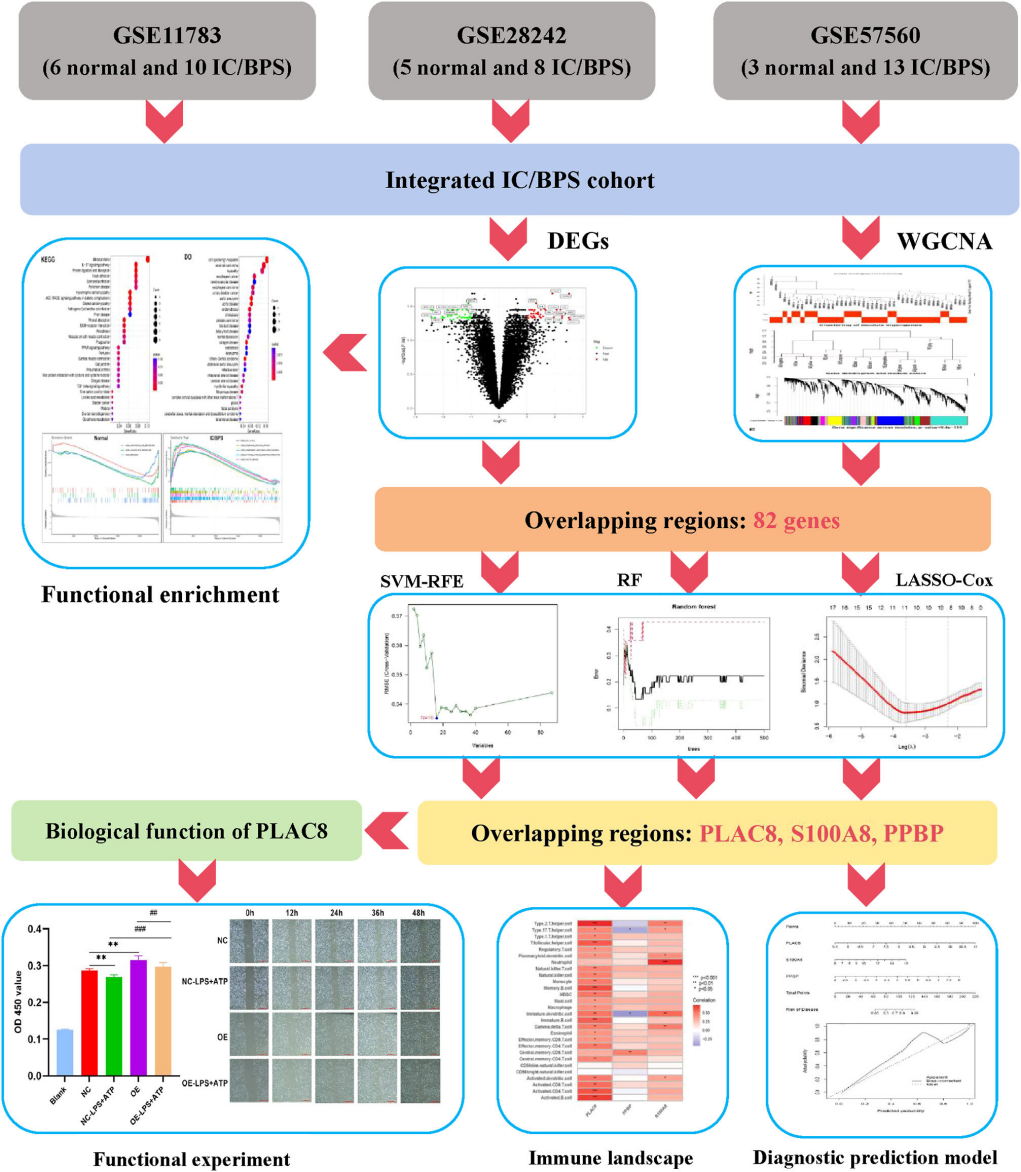
The flowchart depicting the investigation procedure. IC/BPS, interstitial cystitis/painful bladder syndrome; DEGs, Differentially expressed genes; WGCNA, weighted gene co-expression network analysis; RF, random forest; LASSO, loss absolute shrinkage and selection operator; SVM-RFE, support vector machine -recursive feature elimination.

**Figure 2:**
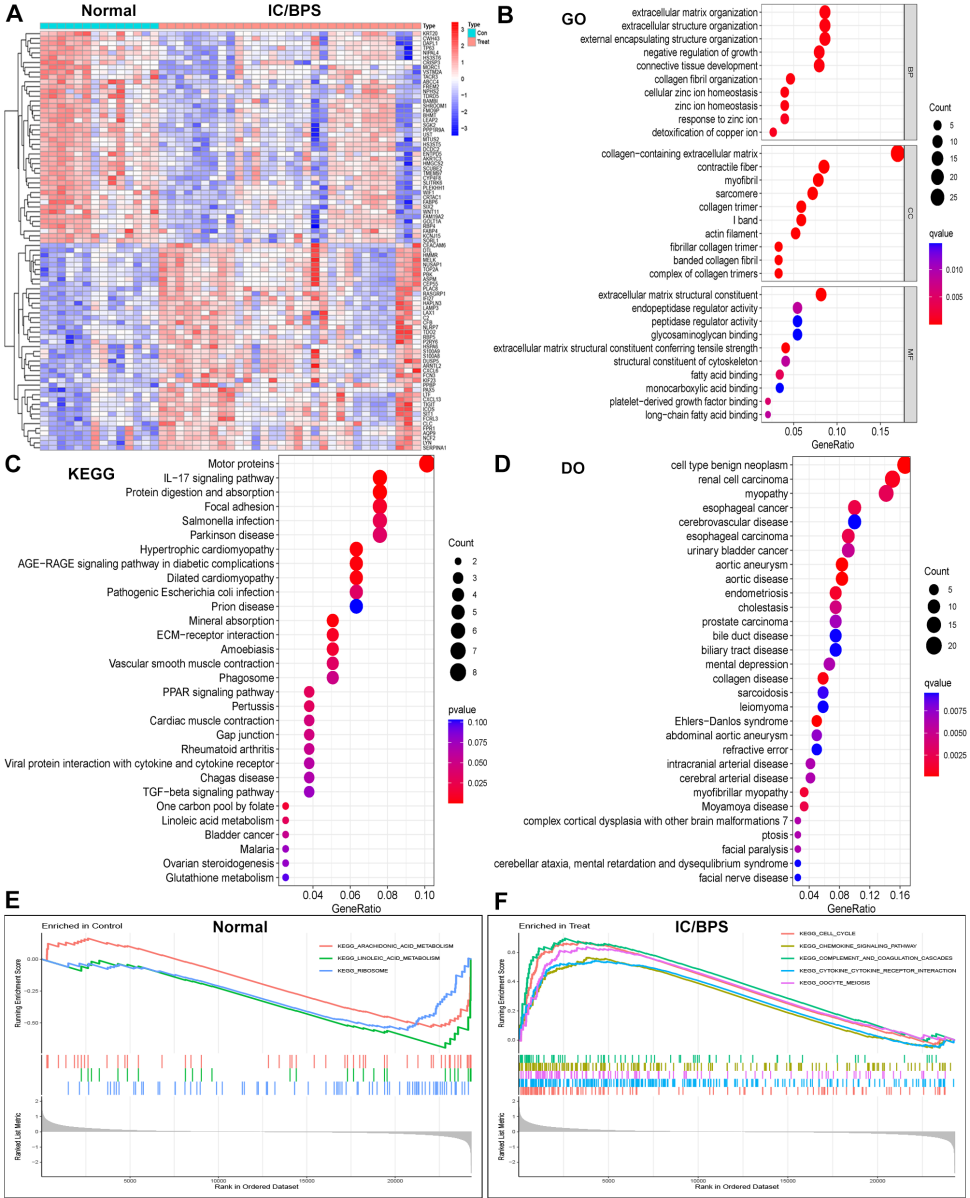
Identification of DEGs in IC/BPS patients and functional enrichment analysis. **(A)** Heatmap showing up-regulated or down-regulated DEGs in IC/BPS samples compared to normal samples (bule: down-regulated; red: up-regulated). **(B)** GO analysis of these DEGs in IC/BPS patients. **(C)** KEGG analysis of these DEGs in IC/BPS patients. **(D)** DO analysis of these DEGs in IC/BPS patients. **(E)** GSEA was performed in normal patients. **(F)** GSEA was performed in IC/BPS patients. IC/BPS, interstitial cystitis/painful bladder syndrome; DEGs, Differentially expressed genes; GO, Gene Ontology; KEGG, Kyoto Encyclopedia of Genes and genes Genomes; DO, Disease Ontology; GSEA, Gene Set Enrichment Analysis.

We acquired three IC/BPS datasets, GSE11783, GSE28242, and GSE57560, from the GEO database, comprising 14 normal samples and 31 IC/BPS samples. Our data analysis process is shown in **Figure 1**. A total of 87 DEGs were identified, comprising 43 up-regulated genes and 44 down-regulated genes (**Figure 2A**; **Supplementary Table 1**). To elucidate the biological functions of these DEGs, we conducted functional enrichment analysis. The results of GO analysis indicated that these DEGs were significantly enriched in processes such as extracellular matrix organization, connective tissue development, collagen fibril organization, collagen-containing extracellular matrix, myofibril, contractile fiber, extracellular matrix structural constituent, peptidase regulator activity, and endopeptidase regulator activity (**Figure 2B**; **Supplementary Table 2**). These biological functions involve the changes of extracellular matrix tissue, connective tissue development, collagen fiber tissue and other factors, which are one of the pathological features of IC/BPS. Furthermore, KEGG analysis unveiled the predominant involvement of DEGs in pathways related to the IL-17 signaling pathway, AGE-RAGE signaling pathway in diabetic complications, and TGF-beta signaling pathway (**Figure 2C** and **Supplementary Table 3**). Abnormal activation of these signaling pathways is a key step in the pathogenesis of interstitial cystitis, exacerbating bladder inflammation and injury by promoting local inflammatory responses and infiltration of immune cells. DO analysis revealed associations with various medical conditions, including cell type benign neoplasm, renal cell carcinoma, myopathy, esophageal cancer, cerebrovascular disease, esophageal carcinoma, urinary bladder cancer, aortic aneurysm, aortic disease, endometriosis, cholestasis, and prostate carcinoma (**Figure 2D**; **Supplementary Table 4**). GSEA was performed on both normal and IC/BPS samples to explore signaling pathways (**Supplementary Table 5**). In the control group, the top three terms included arachidonic acid metabolism, linoleic acid metabolism, and ribosome (**Figure 2E**). In the treatment group, the top five terms comprised cell cycle, chemokine signaling pathway, complement and coagulation cascades, cytokine-cytokine receptor interaction, and oocyte meiosis (**Figure 2F**). The interaction and abnormal activation of these signaling pathways together drive the development of chronic inflammation, fibrosis, and bladder dysfunction of IC/BPS, providing potential targets for treatment.

### 3.2 Identification of key genes by WGCNA analysis in IC/BPS patients

**Figure 3:**
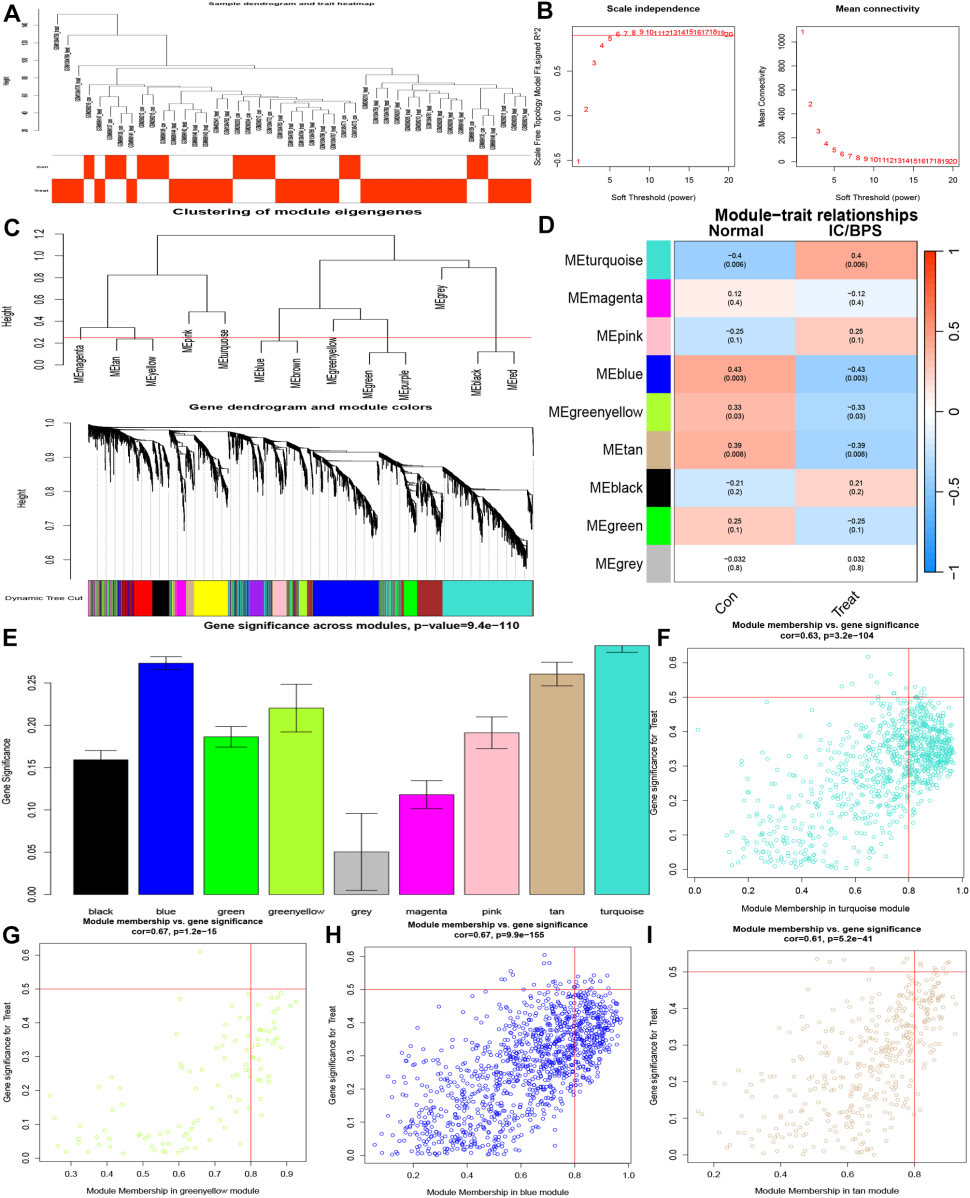
Identification of key genes by WGCNA analysis in IC/BPS patients. **(A)** Clustering dendrogram of 45 samples. **(B)** Analysis of network topology for various soft thresholds (β). **(C)** A dendrogram of the differentially expressed genes clustered based on different metrics. **(D)** Module-trait relationships. **(E)** Distribution of average gene significance and errors in the modules. **(F)** Associations between turquoise module membership and gene importance is depicted in a scatter plot. **(G)** Associations between greenyellow module membership and gene importance is depicted in a scatter plot. **(H)** Associations between blue module membership and gene importance is depicted in a scatter plot. **(I)** Associations between tan module membership and gene importance is depicted in a scatter plot. IC/BPS, interstitial cystitis/painful bladder syndrome; WGCNA, weighted gene co-expression network analysis.

Subsequently, we delved into the identification of key pathogenic genes in IC/BPS through the WGCNA method. The IC/BPS dataset was stratified into two distinct groups—comprising the control group (normal samples) and the treatment group (IC/BPS samples)—with no discernible outliers (**Figure 3A**). Employing a scale-free R^2^ value of 0.95, we ascertained the soft thresholding capability of WGCNA, ultimately selecting 6 as the soft thresholding power β (**Figure 3B**). Subsequent to employing average link hierarchy clustering and evaluating soft threshold capability, we identified 9 modules, with a particular focus on the turquoise, blue, greenyellow, and tan modules due to their significant associations with IC/BPS (**Figures 3C–E**). The correlation between these selected modules and IC/BPS is visually represented in the scatter plot (**Figures 3F–I**). A comprehensive total of 2612 genes exhibited significant associations with IC/BPS in terms of gene significance and functional modules (**Supplementary Table 6**).

### 3.3 Screening and verification of diagnostic markers by bioinformatics and machine-learning algorithm in IC/BPS patients

**Figure 4:**
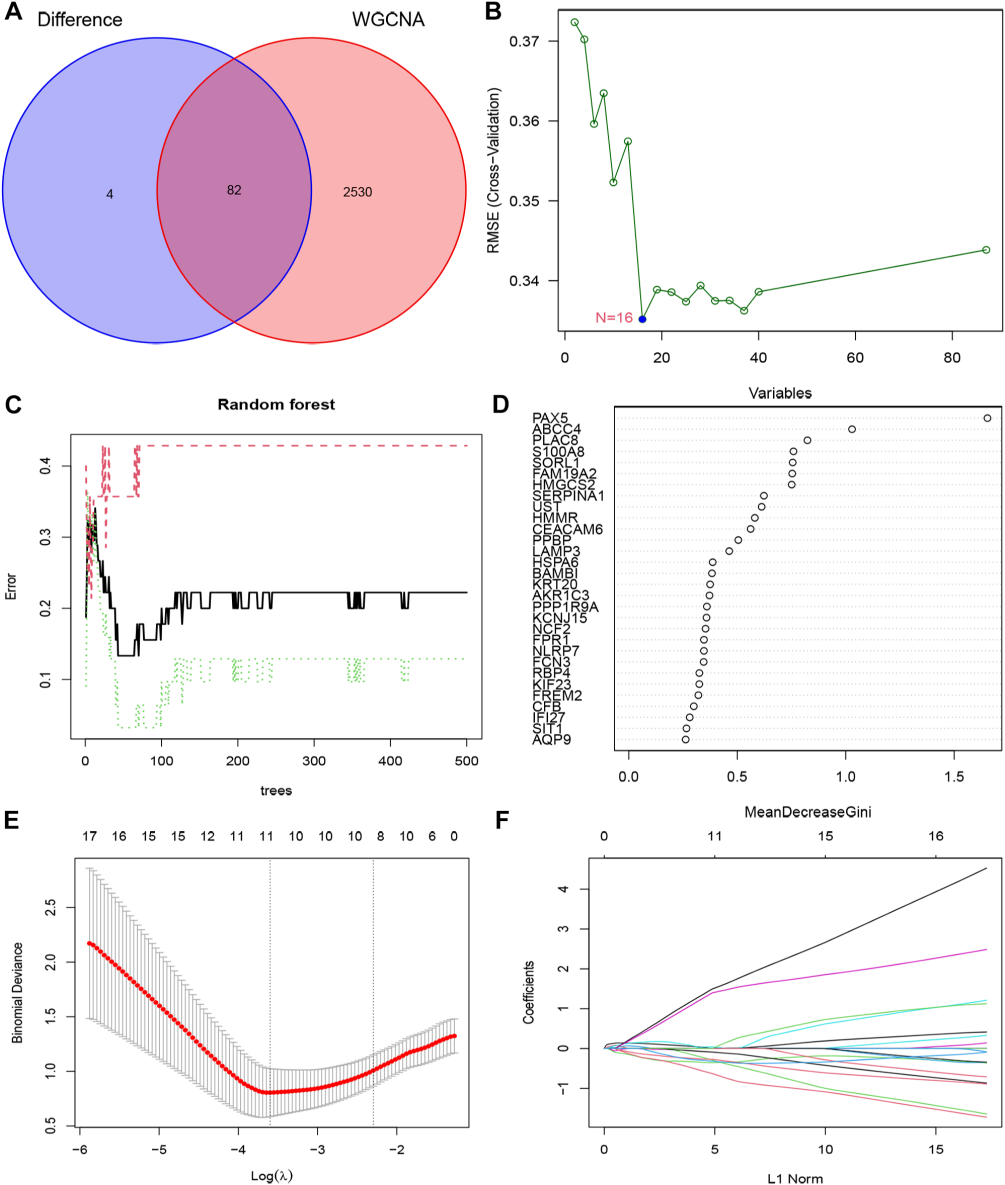
Screening key genes is based on biological analysis and machine-learning algorithms in IC/BPS patients. **(A)** The overlapping regions of key module genes and DEGs. **(B)** Screening of key genes was based on SVM-RFE algorithm. **(C, D)** Screening of key genes was based on RF algorithm. **(E, F)** Screening of key genes was based on LASSO regression analysis. IC/BPS, interstitial cystitis/painful bladder syndrome; DEGs, Differentially expressed genes; RF, random forest; LASSO, loss absolute shrinkage and selection operator; SVM-RFE, support vector machine -recursive feature elimination.

In order to enhance the precision of data processing, we employed a Venn diagram to identify the overlapping genes between DEGs and key module genes in IC/BPS patients. This analysis revealed a total of 82 genes within the overlapping regions (**Figure 4A**; **Supplementary Table 7**). Subsequently, for the identification of potential diagnostic markers in IC/BPS patients, we applied three machine-learning algorithms to pinpoint crucial genes. Initially, the SVM-RFE algorithm identified 16 key genes in IC/BPS patients (**Figure 4B**). Following this, the RF algorithm, coupled with the distinctive characteristics of IC/BPS patients, selected 12 key genes (**Figures 4C, D**). Lastly, utilizing LASSO regression analysis, we identified 7 key genes from statistically significant univariate variables in IC/BPS patients (**Figures 4E, F**).

**Figure 5:**
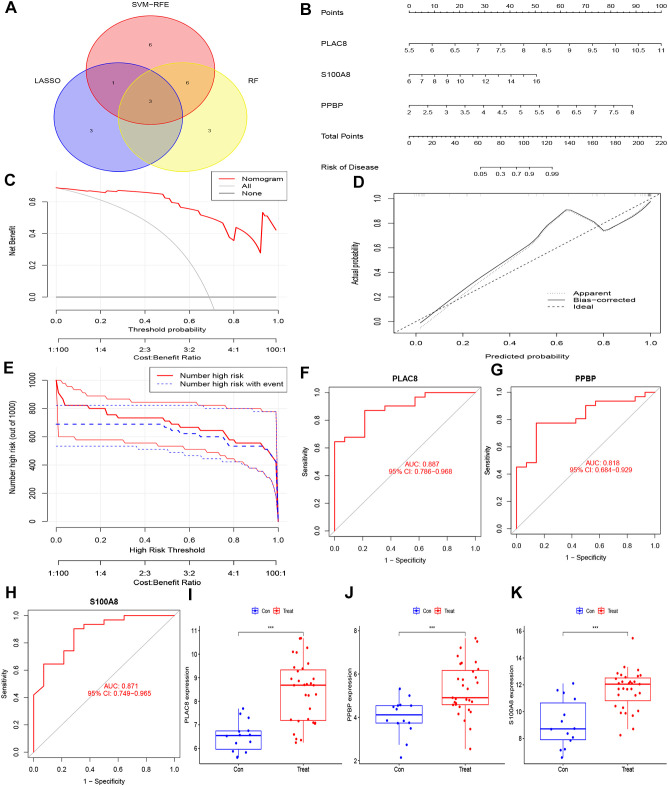
Hub genes for pediatric sepsis diagnosis in IC/BPS patients. **(A)** Venn diagram showed the intersection of diagnostic markers obtained by the three algorithms. **(B)** Nomogram is used to predict the occurrence of IC/BPS. **(C)** DCA curves of the diagnostic efficacy verification; **(D)** Calibration curve of the diagnostic efficacy verification; **(E)** Clinical impact curve of the diagnostic efficacy verification. **(F)** The ROC curve of PLAC8 in IC/BPS dataset; **(G)** The ROC curve of PPBP in IC/BPS dataset. **(H)** The ROC curve of S100A8 in IC/BPS dataset. **(I)** Boxplot showed the expression of PLAC8 between IC/BPS group and control group. **(J)** Boxplot showed the expression of PPBP between IC/BPS group and control group. **(K)** Boxplot showed the expression of S100A8 between IC/BPS group and control group. IC/BPS, interstitial cystitis/painful bladder syndrome; DCA, decision curve analysis; CIC, clinical impact curve; ROC, receiver operating characteristic. ***, P < 0.001.

The integration of the predictions from these three machine-learning algorithms highlighted three key genes: PLAC8, S100A8, and PPBP (**Figure 5A**). To assess the clinical significance of these key genes, we constructed a nomogram model for diagnosing IC/BPS based on these predictive genes (**Figure 5B**). Decision curve analysis (DCA), calibration curve, and clinical impact curve (CIC) results demonstrated a favorable clinical benefit of our nomogram model (**Figures 5C–E**). Additionally, ROC curve analysis indicated that PLAC8 (AUC: 0.887), S100A8 (AUC: 0.818), and PPBP (AUC: 0.871) may serve as valuable biomarkers for IC/BPS patients (**Figures 5F–H**). Furthermore, the mRNA expression levels of PLAC8, S100A8, and PPBP were significantly upregulated in IC/BPS patients compared to normal patients (**Figures 5I–K**).

### 3.4 Immune cell landscape and its associations with diagnostic genes in IC/BPS patients

**Figure 6:**
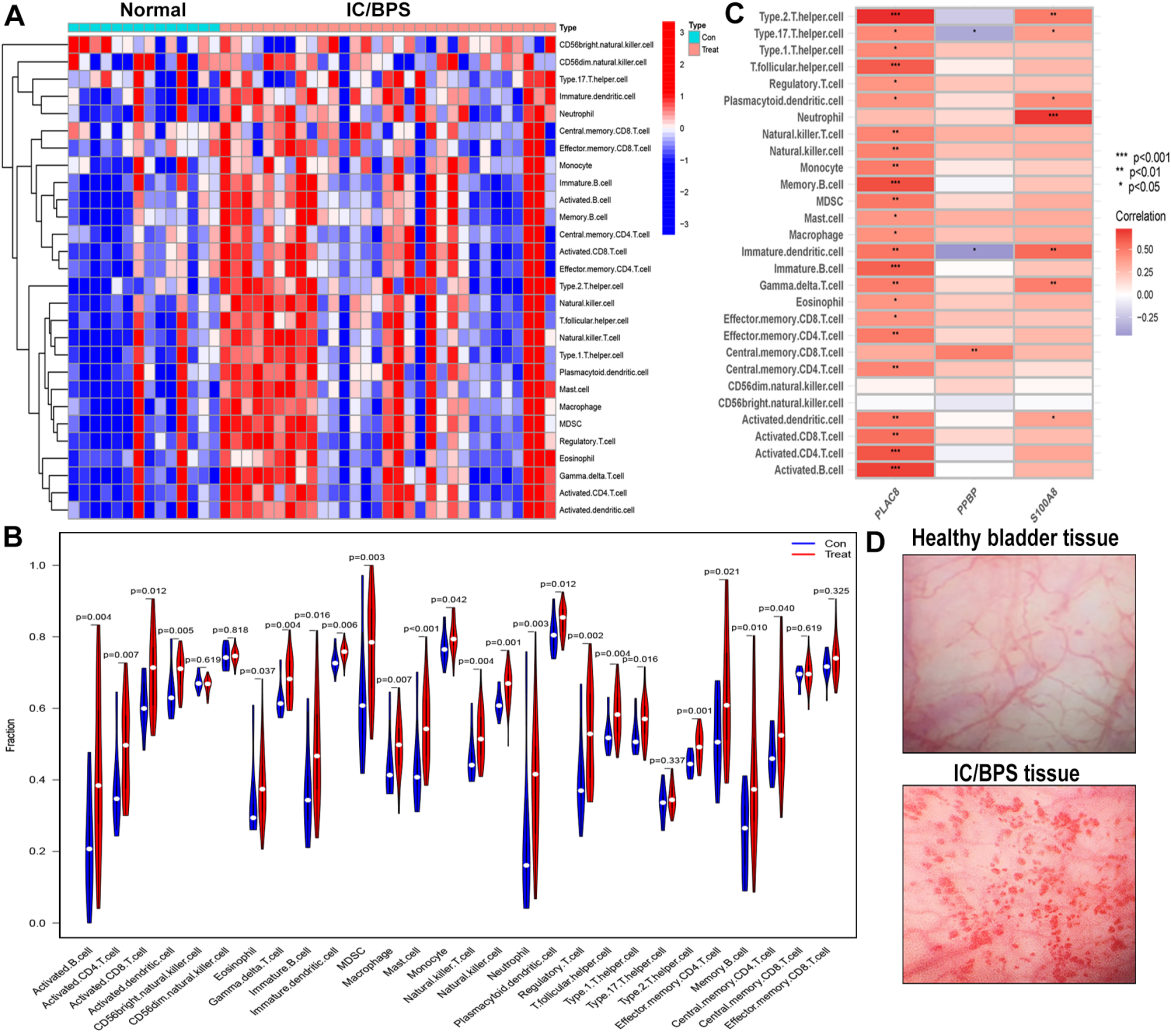
Evaluation and visualization of immune cell infiltration in IC/BPS cohort. **(A)** Heat maps confirm that most IC/BPS samples have higher immune cells compared to normal samples. **(B)** The immune landscape reveal the infiltration levels of 23 types of immune cells in IC/BPS samples were significantly higher than those in normal samples. **(C)** Correlation between three hub genes and immune cell in IC/BPS dataset. **(D)** Representative cystoscopic results of healthy bladder tissue and IC/BPS tissue with Hunner’s lesions in one case. Diagnosis is made via cystoscopy and confirmed by biopsy. IC/BPS, interstitial cystitis/painful bladder syndrome. *P < 0.05; **P < 0.01; ***P < 0.001.

We employed the ssGSEA algorithm to assess the landscape of immune cell infiltration in IC/BPS compared to normal samples. Our findings revealed a notable increase in the infiltration levels of 28 types of immune cells in the majority of IC/BPS samples as opposed to normal samples (**Figure 6A**). Specifically, the infiltration levels of 23 types of immune cells in IC/BPS samples were significantly higher than those in normal samples, encompassing Activated B cells, Activated CD4 T cells, Activated CD8 T cells, Activated dendritic cells, Eosinophils, Gamma delta T cells, Immature B cells, Immature dendritic cells, MDSCs, Macrophages, Mast cells, Monocytes, Natural killer T cells, Natural killer cells, Neutrophils, Plasmacytoid dendritic cells, Regulatory T cells, T follicular helper cells, Type 1 T helper cells, Type 2 T helper cells, Effector memory CD4 T cells, Memory B cells, and Central memory CD4 T cells (**Figure 6B**).

**Figure 7:**
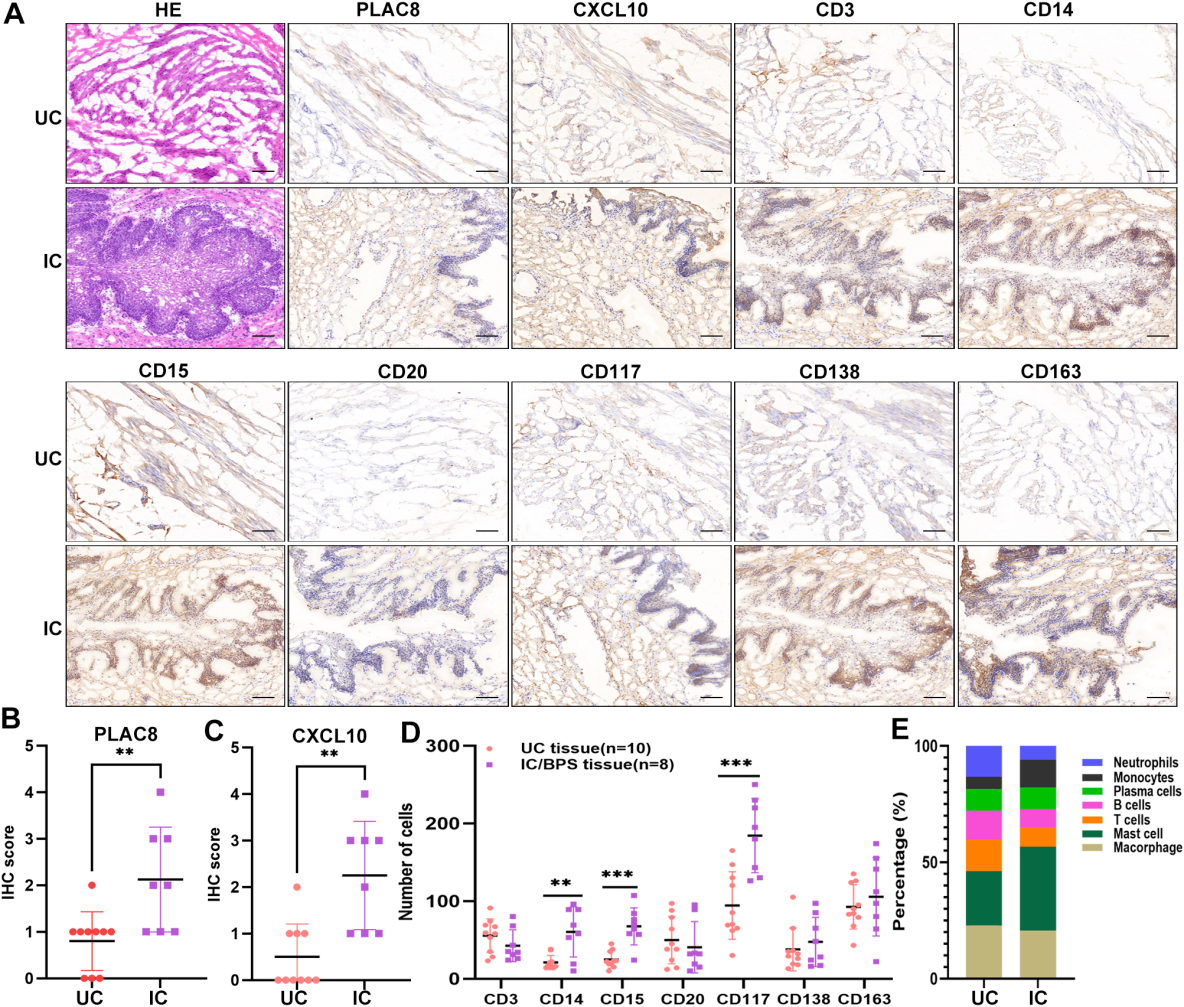
Verification of PLAC8 expression and immune cell infiltration in verified cohort. **(A)** Representative results of protein expression levels of PLAC8, CXCL10 and 7 immune cell markers in IC/BPS tissue and UC tissue (Microscale: 100 μm). **(B)** The IHC score of PLAC8 in IC/BPS tissues and UC tissues. **(C)** The IHC score of CXCL10 in IC/BPS tissues and UC tissues. **(D)** Number of positive cells for seven immune cell marker in IC/BPS tissues and UC tissues. **(E)** The relative proportions of seven immune cell marker’s protein expression in IC/BPS tissues and UC tissues. IC/BPS, interstitial cystitis/painful bladder syndrome; UC, unaffected control; IHC, Immunohistochemistry. **P < 0.01; ***P < 0.001.

Furthermore, our analysis indicated significant positive associations between three key genes and multiple immune cells in IC/BPS patients, particularly PLAC8 (**Figure 6C**). To validate the role of these key genes and immune cells in IC/BPS patients, we gathered 18 tissue samples (10 UC samples and 8 IC/BPS samples) for subsequent analysis, confirmed through cystoscopy and tissue biopsy (**Figure 6D**). There was no significant difference in clinical features between the two groups (**Table 2**). In IC/BPS tissues, we observed significantly upregulated expression of PLAC8, CXCL10, CD14 (Monocytes), CD15 (Neutrophils), and CD117 (Mast cells) compared to UC tissues (**Figures 7A–D**). The relative proportions of each immune cell marker’s protein expression in the UC and IC/BPS cohorts exhibited distinctions, with a notable increase in mast cells and monocytes in IC/BPS tissues (**Figure 7E**).

**Table 2:** The clinical features data from UC samples and IC/BPS samples.

Variable	Total (n = 18)	Group		*P*-value
		IC/BPS (n = 8)	UC (n = 10)	
Age, M (Q_1_, Q_3_)	60.50 (55.50 - 67.75)	57.00 (53.75 - 62.00)	65.50 (57.50 - 74.50)	0.118
BMI, M (Q_1_, Q_3_)	23.44 (22.59 - 24.35)	23.72 (21.64 - 24.28)	23.38 (22.73 - 24.77)	0.859
Hb, M (Q_1_, Q_3_)	122.00 (119.00 - 130.00)	122.00 (119.00 - 128.00)	122.00 (115.50 - 130.00)	0.859
WBC, M (Q_1_, Q_3_)	5.12 (4.49 - 6.47)	4.67 (4.07 - 5.42)	5.66 (4.75 - 6.47)	0.315
NLR, M (Q_1_, Q_3_)	1.61 (1.02 - 2.29)	1.05 (0.96 - 1.30)	2.08 (1.68 - 2.49)	0.056
LMR, M (Q_1_, Q_3_)	3.37 (2.72 - 5.13)	3.20 (2.09 - 5.33)	3.75 (2.85 - 4.61)	0.515
ALC, M (Q_1_, Q_3_)	1.67 (1.56 - 1.88)	1.79 (1.66 - 1.99)	1.59 (1.37 - 1.80)	0.351
Gender, n (%)				0.092
Female	14 (77.78)	8 (100.00)	6 (60.00)	
Male	4 (22.22)	0 (0.00)	4 (40.00)	
History of urinary surgery, n (%)				0.153
No	9 (50)	6 (75.00)	3 (30.00)	
Yes	9 (50)	2 (25.00)	7 (70.00)	
Diabetes, n (%)				1.000
No	16 (88.89)	7 (87.50)	9 (90.00)	
Yes	2 (11.11)	1 (12.50)	1 (10.00)	
Hypertension, n (%)				1.000
No	13 (72.22)	6 (75.00)	7 (70.00)	
Yes	5 (27.78)	2 (25.00)	3 (30.00)	
Smoke history, n (%)				0.216
No	15 (83.33)	8 (100.00)	7 (70.00)	
Yes	3 (16.67)	0 (0.00)	3 (30.00)	
Drinking history, n (%)				0.216
No	15 (83.33)	8 (100.00)	7 (70.00)	
Yes	3 (16.67)	0 (0.00)	3 (30.00)	

UC, unaffected control; IC/BPS, interstitial cystitis/painful bladder syndrome; BMI, body mass index; Hb, Hemoglobin; WBC, white blood cell; NLR, neutrophil to lymphocyte ratio; LMR, Lymphocyte-to-Monocyte Ratio; ALC, absolute lymphocyte count.

### 3.5 Effects of PLAC8 expression on the biological function of urothelial cells *in vitro and in vivo*

**Figure 8:**
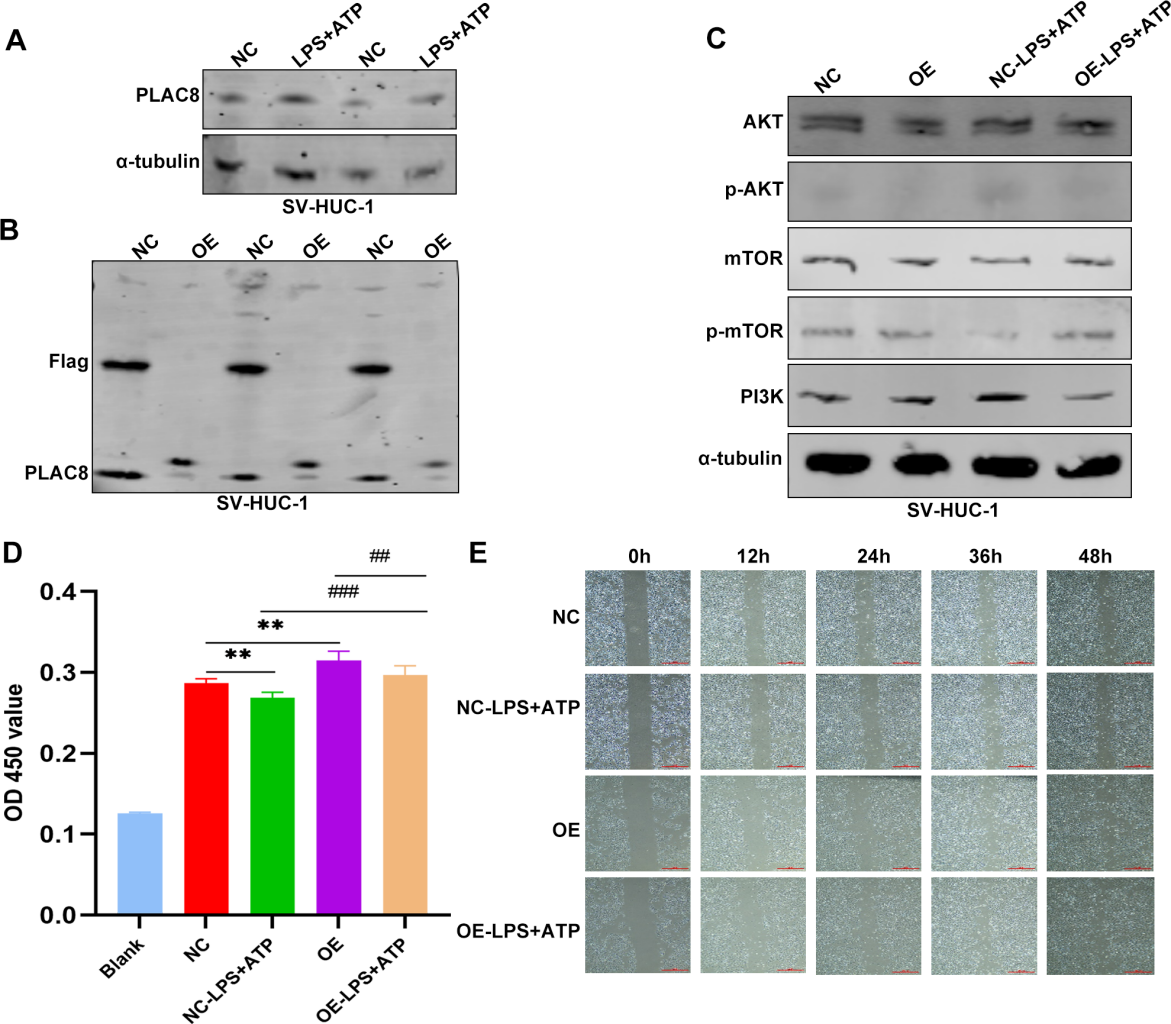
The expression and biological functions of PLAC8 in IC/BPS were further validated through *in vitro* experiments. **(A)** Representative results of the expression level of PLAC8 protein in inflammatory cell model was detected by western blot. **(B)** Representative results of PLAC8 overexpression in SV-HUC-1 cells by western blot. **(C)** Representative results of AKT/mTOR/PI3K pathway marker proteins were detected by western blot in SV-HUC-1 cells. **(D)** Proliferation of SV-HUC-1 cells was detected by cell counting kit-8. **(E)** Representative results of migration ability were measured by wound healing assay in SV-HUC-1 cells (Microscale: 500 μm). OD, optical density; NC, negative control; OE, over expression. **P < 0.01; ##P < 0.01; ###P < 0.001.

PLAC8 protein expression levels were significantly upregulated in inflammatory cell models induced by LPS and ATP compared to controls (**Figure 8A**). Following transfection, PLAC8 was overexpressed in SV-HUC-1 cells, as confirmed by western blot analysis, compared to control cells (**Figure 8B**). We observed that the expression of key proteins in the AKT/mTOR/PI3K signaling pathway—such as AKT, mTOR, and PI3K—was inhibited in PLAC8-overexpressing cells compared to the control group, including in the inflammatory cell models (**Figure 8C**). PLAC8 overexpression significantly enhanced the proliferative capacity of SV-HUC-1 cells compared to untreated cells (**Figure 8D**). However, a wound healing assay revealed that PLAC8 had no significant effect on the migration ability of SV-HUC-1 cells, including in the inflammatory cell models (**Figure 8E**).

## 4 Discussion

IC/BPS is a chronic condition characterized by pelvic pain, discomfort, and urinary symptoms. Research efforts focused on IC/BPS aim to comprehend its etiology, enhance diagnostic methods, and devise effective treatments (6). Currently, it is noteworthy to recognize that inflammation and immune cell response constitute key features in IC/BPS patients. In this study, the integration of bioinformatics and machine-learning algorithms led to the identification of three upregulated DEGs. The immune landscape analysis unveiled a robust positive association between PLAC8 and various immune cells. Our findings suggest that PLAC8 may serve as a diagnostic biomarker and a potential target for modulating immune responses in individuals with IC/BPS.

In IC/BPS, inflammation is commonly characterized as non-infectious, indicating that it is not attributed to bacteria or other pathogens typically associated with urinary tract infections (37). The diagnostic process is often intricate due to the variability in IC/BPS symptoms and their overlap with other urinary disorders. Pathological diagnosis reports in IC/BPS are typically obtained only after transurethral resection of Hunner’s lesion, which may introduce bias and cause delays in diagnosis. A comprehensive approach to IC/BPS diagnosis, involving clinical evaluation, laboratory tests, and imaging studies, is crucial (38). Ongoing efforts to identify diagnostic biomarkers and develop urine tests are focused on establishing non-invasive methods for diagnosing IC/BPS. For example, chemokines such as CXCL-10 may serve as valuable biomarkers for both diagnosing IC/BPS and assessing its clinical characteristics. In this study, we reported a novel diagnostic biomarker for identifying patients with IC/BPS. Differential analysis identified 43 up-regulated DEGs and 44 down-regulated DEGs in the IC/BPS cohort. Functional enrichment analysis indicated that these genes were associated with extracellular matrix regulation, the IL-17 signaling pathway, and the TGF-beta signaling pathway. To assess the predictive value of these genes in IC/BPS patients, we constructed a nomogram model, demonstrating their efficacy in accurately predicting the occurrence of IC/BPS. Our findings align with several prior studies that identified novel diagnostic biomarkers through integrated approaches (12, 13, 18). Unfortunately, due to a lack of available data, our model could not be validated in independent IC/BPS datasets.

Chronic inflammation and abnormal immune responses are implicated in the occurrence and progression of IC/BPS (11, 39). Employing bioinformatics analysis and machine-learning algorithms, our study identified three hub genes—PLAC8, S100A8, and PPBP—that may be involved in the disease progression of IC/BPS patients. Previous reports indicate that PLAC8 is a multifunctional immunomodulator that was originally discovered in the placenta and has been shown to be valuable in multiple immune responses (40). PLAC8 regulates inflammatory responses and activation of immune cells, affecting the immune system’s response to pathogens or other exogenous stimuli (41, 42). For example, PLAC8 has been observed to mediate T cell regulation in clearing genital chlamydia infection (43). In chronic inflammation, rheumatoid arthritis, and autoimmune diseases, PLAC8 expression is often elevated, potentially exacerbating or modulating the inflammatory response by influencing the function of immune cells and the levels of secreted cytokines (41, 44, 45). Our study revealed a significantly higher expression level of PLAC8 protein than in normal healthy tissues, indicating its potential relevance to the pathogenic process of IC/BPS. S100A8/S100A9 is released by neutrophils and monocytes and is considered to be a key regulator involved in a variety of inflammatory responses (46, 47). Current studies confirm its potential use as a predictive biomarker for inflammatory developments such as juvenile rheumatoid arthritis, autoimmune synovitis, and autoimmune diseases (48–50). Moreover, blocking soluble S100A8/A9 secretion may offer an inspiring therapeutic strategy for inflammatory diseases (51). While these findings underscore the crucial role of S100A8 in inflammatory processes, its specific value in patients with IC/BPS warrants further evaluation. PPBP, identified as a potent neutrophil activator, has been shown to activate substance metabolism and synthesis in various cells (52). Numerous studies associate PPBP disorders with inflammatory conditions like acute lung injury and chronic obstructive pulmonary disease (52, 53). Zhang et al. reported PPBP as a marker of podocyte injury in diabetic nephropathy (54). PPBP may participate in immune response regulation by inducing chronic inflammation or Th2 polarization, suggesting its potential involvement in mediating immune response processes in IC/BPS patients (55). Collectively, these findings propose that the three identified candidate genes—PLAC8, S100A8, and PPBP—may play crucial roles in the disease progression of IC/BPS patients, serving as potential diagnostic markers and therapeutic targets.

Research indicates that the immune system’s response in IC/BPS may be triggered by various factors, including infection, injury, or other unknown elements (1). Several immune cells, such as mast cells and T lymphocytes, are implicated in the pathogenesis of IC/BPS (56). Mast cells release inflammatory mediators that contribute to pain and inflammation, while T lymphocytes play a role in immune responses (11). Abnormalities in the function of these immune cells may contribute to the chronic inflammation observed in IC/BPS patients. In our study, we utilized the ssGSEA algorithm and IHC staining to evaluate the immune cell landscapes in IC/BPS patients. Notably, IC/BPS patients exhibited a significantly higher proportion of innate and adaptive immune cell infiltration compared to normal patients (11, 12). We observed a significant positive correlation between PLAC8 expression and various immune cell types, including T cells, B cells, macrophages, dendritic cells, and mast cells. These findings suggest that PLAC8 may play a role in initiating immune responses, maintaining immune tolerance, and regulating inflammatory responses by modulating the function of multiple immune cell types. Neutrophils, a type of white blood cell, play a crucial role in the body’s immune response, particularly in the early stages of inflammation and infection (57). Neutrophils can directly contribute to epithelial damage in urothelial tissue through the release of a variety of harmful mediators, including proteases, reactive oxygen species (ROS), and inflammatory cytokines (58, 59). Furthermore, the prolonged presence of neutrophils and their ongoing release of inflammatory mediators can contribute to the persistence and exacerbation of the inflammatory response (60). Some studies have suggested that inflammation may contribute to the pathophysiology of interstitial cystitis, and neutrophils could be involved in this inflammatory response (59, 61). However, the precise mechanisms and the extent of neutrophil involvement in interstitial cystitis are not fully understood. It’s important to note that the understanding of the relationship between immune cells and interstitial cystitis is still evolving, and more research is needed to fully elucidate the underlying mechanisms.

The PI3K/Akt/mTOR signaling pathway is a crucial signal transduction pathway in mammals, playing a vital role in various biological processes (62). For instance, the activity of this pathway is essential in tissue repair and inflammatory diseases (63, 64). Activation of the PI3K/Akt/mTOR signaling pathway can inhibit autophagy and promote inflammation, whereas its inhibition can enhance autophagy and reduce inflammation (65, 66). In this study, activation of the PI3K/Akt/mTOR signaling pathway was observed in inflammatory cell models, while PLAC8 overexpression inhibited this activation, suggesting a potential therapeutic role for PLAC8 in the inflammatory process of urothelial cells. The possibility of downregulating PLAC8 to modulate the PI3K/Akt/mTOR signaling pathway and suppress the inflammatory response presents a promising avenue for the treatment and prevention of IC/BPS. Although further clinical and experimental studies are needed to confirm our findings, our results offer valuable insights into IC/BPS biomarkers and lay a solid foundation for future research.

## 5 Conclusions

In summary, we harnessed genomic data to integrate bioinformatics analysis and machine-learning algorithms, unveiling molecular characteristics and immune landscapes specific to IC/BPS patients. The three hub genes, PLAC8, S100A8, and PPBP, are significantly associated with various immune cell types in IC/BPS. Among them, PLAC8 is a promising diagnostic biomarker that modulates the PI3K/Akt/mTOR signaling pathway in patients with IC/BPS, which provides new insights into the future diagnosis of IC/BPS.

## Data Availability

The raw data supporting the conclusions of this article will be made available by the authors, without undue reservation.
