# Supplementary material for: Identification and validation of immune and diagnostic biomarkers for interstitial cystitis/painful bladder syndrome by integrating bioinformatics and machine-learning
Source: Front Immunol. 2025 Jan 23;16:1511529. doi: 10.3389/fimmu.2025.1511529 (PMC11799275; doi:10.3389/fimmu.2025.1511529)
Supplement: Supplementary file 1 [file Table1.docx]

**Table S1 Differentially expressed genes** **in the IC/BPS datasets.**

| **id** | **logFC** | **AveExpr** | **t** | **P.Value** | **adj.P.Val** | **B** |
| --- | --- | --- | --- | --- | --- | --- |
| PLAC8 | 1.908099896 | 7.815849744 | 5.317593892 | 2.94E-06 | 0.020471787 | 4.373940826 |
| S100A8 | 2.511776196 | 10.85618277 | 5.15491857 | 5.11E-06 | 0.020471787 | 3.884269295 |
| IFI27 | 1.332960158 | 9.451673694 | 4.811581379 | 1.62E-05 | 0.028700424 | 2.861478926 |
| ABCC4 | -1.104029689 | 6.786650381 | -4.775665246 | 1.83E-05 | 0.028700424 | 2.755503168 |
| CFB | 1.633851705 | 8.010616629 | 4.663415222 | 2.65E-05 | 0.034708631 | 2.42574349 |
| PAX5 | 1.083386632 | 4.364580181 | 4.574574845 | 3.55E-05 | 0.035517005 | 2.166425312 |
| NIPAL4 | -1.677530669 | 7.502448666 | -4.484378673 | 4.78E-05 | 0.035517005 | 1.90478755 |
| CLC | 2.197374814 | 5.890396464 | 4.393598786 | 6.42E-05 | 0.035517005 | 1.64324669 |
| HMGCS2 | -2.42827935 | 7.559497577 | -4.388729758 | 6.52E-05 | 0.035517005 | 1.629272012 |
| KIF23 | 1.163549702 | 5.012396056 | 4.378474321 | 6.74E-05 | 0.035517005 | 1.599855906 |
| FAM19A2 | -1.637367427 | 5.262620616 | -4.309411774 | 8.43E-05 | 0.035517005 | 1.40241683 |
| SORL1 | -1.170075861 | 11.29321485 | -4.253789347 | 0.000100793 | 0.035517005 | 1.244258937 |
| KCNJ15 | -1.312649947 | 7.374420235 | -4.231213122 | 0.000108361 | 0.035517005 | 1.180291371 |
| CXCL6 | 2.07438196 | 5.76767042 | 4.226452487 | 0.000110027 | 0.035517005 | 1.16681956 |
| UST | -1.14234753 | 6.503863674 | -4.224321937 | 0.00011078 | 0.035517005 | 1.160792386 |
| PBK | 1.662329971 | 3.753001344 | 4.204955093 | 0.000117862 | 0.035517005 | 1.106060014 |
| WIF1 | -2.375845098 | 4.990555773 | -4.193371716 | 0.000122306 | 0.035517005 | 1.073372158 |
| SLITRK6 | -1.821458909 | 8.205068392 | -4.188227505 | 0.000124331 | 0.035517005 | 1.058866948 |
| TOP2A | 1.497686025 | 4.230186366 | 4.17483432 | 0.000129759 | 0.035517005 | 1.021135556 |
| ARNTL2 | 1.280626759 | 6.505332025 | 4.164254796 | 0.000134209 | 0.035517005 | 0.99136536 |
| TP63 | -1.497076793 | 6.015432721 | -4.161172955 | 0.000135533 | 0.035517005 | 0.982698997 |
| HAPLN3 | 1.192654889 | 7.043443425 | 4.154812892 | 0.000138305 | 0.035517005 | 0.964822291 |
| PPP1R9A | -1.455079139 | 5.754629649 | -4.153627024 | 0.000138828 | 0.035517005 | 0.961490318 |
| FCN3 | 1.13492394 | 4.820645181 | 4.152051061 | 0.000139526 | 0.035517005 | 0.957062882 |
| DTL | 1.243077082 | 4.697044985 | 4.088427809 | 0.000170738 | 0.037256527 | 0.778904374 |
| LAMP3 | 1.398674728 | 8.217484212 | 4.057682428 | 0.000188155 | 0.038849655 | 0.693226261 |
| S100A9 | 2.038915712 | 9.258773458 | 4.053916789 | 0.000190403 | 0.038849655 | 0.682751565 |
| NLRP7 | 1.326630184 | 3.906165503 | 4.031479316 | 0.000204349 | 0.040131502 | 0.620425342 |
| ASPM | 1.231206954 | 4.021219195 | 4.020843402 | 0.0002113 | 0.04053426 | 0.590933654 |
| CEP55 | 1.377809819 | 4.235273443 | 4.018947403 | 0.000212563 | 0.04053426 | 0.585679923 |
| RASGRP1 | 1.156525684 | 7.245470348 | 4.016596729 | 0.000214139 | 0.04053426 | 0.579167812 |
| TDRD5 | -1.009550614 | 3.066747058 | -4.010138875 | 0.000218528 | 0.040872514 | 0.561286103 |
| HS3ST6 | -1.56226594 | 6.45536787 | -3.998007559 | 0.000227009 | 0.041471297 | 0.527728877 |
| TACR3 | -1.211477548 | 3.512645787 | -3.987977124 | 0.000234261 | 0.041582862 | 0.500016967 |
| ICOS | 1.487509564 | 5.073963473 | 3.967299327 | 0.000249925 | 0.041582862 | 0.4429865 |
| HMMR | 1.411592451 | 4.546905352 | 3.967276939 | 0.000249943 | 0.041582862 | 0.442924823 |
| TMEM97 | -1.824420334 | 9.143614563 | -3.960675885 | 0.000255154 | 0.041582862 | 0.424746756 |
| HS3ST5 | -1.516359026 | 5.416880893 | -3.955510051 | 0.000259306 | 0.041582862 | 0.410530512 |
| KRT20 | -1.697445651 | 4.823741857 | -3.930716107 | 0.000280157 | 0.042927176 | 0.342415119 |
| FREM2 | -1.727461323 | 4.395834095 | -3.926858252 | 0.000283544 | 0.042927176 | 0.331834108 |
| NPHS2 | -1.742991075 | 5.114524912 | -3.92323068 | 0.000286765 | 0.042927176 | 0.32188903 |
| P2RY6 | 1.044004351 | 6.336355659 | 3.912857105 | 0.000296172 | 0.042927176 | 0.293472868 |
| CRISP3 | -1.833551308 | 5.769151185 | -3.883467821 | 0.000324471 | 0.043731326 | 0.213156005 |
| SIT1 | 1.011490873 | 6.329860718 | 3.864961527 | 0.000343612 | 0.043731326 | 0.162725654 |
| MELK | 1.236538366 | 4.465957636 | 3.864781763 | 0.000343803 | 0.043731326 | 0.162236346 |
| AQP9 | 2.266303832 | 6.399096626 | 3.851383316 | 0.000358345 | 0.043731326 | 0.125796556 |
| MORC1 | -1.040594035 | 3.18181816 | -3.831573356 | 0.000380938 | 0.04433269 | 0.072029259 |
| HSPA6 | 1.238234625 | 7.314776021 | 3.825069728 | 0.00038865 | 0.0444662 | 0.054406215 |
| SHROOM1 | -1.406393652 | 7.931804501 | -3.818806174 | 0.00039622 | 0.0444662 | 0.037447255 |
| NUSAP1 | 1.250418888 | 5.311618052 | 3.817250261 | 0.000398123 | 0.0444662 | 0.033236592 |
| TDO2 | 1.455514784 | 3.674066459 | 3.814575412 | 0.000401414 | 0.0444662 | 0.025999751 |
| C2 | 1.141337971 | 7.221563169 | 3.811304921 | 0.000405473 | 0.0444662 | 0.017154708 |
| SCUBE2 | -2.022912049 | 9.854966273 | -3.810040765 | 0.000407053 | 0.0444662 | 0.013736779 |
| LEAP2 | -1.836112679 | 7.549049746 | -3.807342363 | 0.000410445 | 0.0444662 | 0.006442868 |
| NCF2 | 1.577476762 | 7.967461439 | 3.805775845 | 0.000412427 | 0.0444662 | 0.002209636 |
| FPR1 | 1.964588553 | 7.826056295 | 3.787037787 | 0.000436856 | 0.045456555 | -0.048361167 |
| SIX2 | -1.2933013 | 5.680625095 | -3.787014626 | 0.000436888 | 0.045456555 | -0.048423599 |
| WNT11 | -1.060882239 | 4.218518604 | -3.780026492 | 0.000446351 | 0.045834145 | -0.067252254 |
| BHMT | -2.411067096 | 6.107797366 | -3.766209578 | 0.000465647 | 0.046394564 | -0.104430162 |
| SGK2 | -1.156852268 | 5.603830911 | -3.759979662 | 0.000474608 | 0.046394564 | -0.121171475 |
| FABP6 | -1.332306684 | 5.407537826 | -3.756318564 | 0.000479951 | 0.046394564 | -0.13100338 |
| LTF | 2.261458277 | 6.129098312 | 3.741861918 | 0.000501622 | 0.046394564 | -0.169780553 |
| SERPINA1 | 1.486603388 | 7.37416373 | 3.741233006 | 0.000502585 | 0.046394564 | -0.171465806 |
| MTUS2 | -1.054395243 | 5.449448153 | -3.723849944 | 0.00052994 | 0.046394564 | -0.217990244 |
| AKR1C3 | -1.146546059 | 10.10543716 | -3.722096861 | 0.000532777 | 0.046394564 | -0.222676232 |
| RBP5 | 1.040562707 | 6.205639738 | 3.715938853 | 0.000542859 | 0.046394564 | -0.239127826 |
| PLEKHH1 | -1.012843135 | 5.645967676 | -3.713170249 | 0.000547451 | 0.046394564 | -0.246519914 |
| CWH43 | -2.02527858 | 5.558605453 | -3.712235989 | 0.000549009 | 0.046394564 | -0.249013735 |
| PPBP | 1.280118426 | 4.908467537 | 3.70624183 | 0.000559107 | 0.046394564 | -0.265006443 |
| FCRL3 | 1.295515596 | 4.542842752 | 3.705812851 | 0.000559836 | 0.046394564 | -0.266150479 |
| VSTM2A | -1.22634063 | 5.012446651 | -3.695192009 | 0.000578192 | 0.046394564 | -0.294453738 |
| BAMBI | -1.026488266 | 9.834442511 | -3.69270015 | 0.000582581 | 0.046394564 | -0.301088293 |
| TIGIT | 1.28928064 | 5.332502171 | 3.690862207 | 0.000585839 | 0.046394564 | -0.305980349 |
| DUSP5 | 1.49856906 | 7.683706731 | 3.690056852 | 0.000587272 | 0.046394564 | -0.308123572 |
| GOLT1A | -1.958742277 | 6.528416602 | -3.680322221 | 0.000604864 | 0.046394564 | -0.33401076 |
| CEACAM6 | 2.493422221 | 7.175764491 | 3.6768813 | 0.000611203 | 0.046394564 | -0.343152836 |
| FMO9P | -2.299386425 | 5.948652169 | -3.674622998 | 0.000615397 | 0.046394564 | -0.349150481 |
| ENTPD5 | -1.115113248 | 7.20922116 | -3.671325344 | 0.000621572 | 0.046394564 | -0.357905074 |
| CRTAC1 | -1.971017442 | 6.932284968 | -3.669752021 | 0.000624539 | 0.046394564 | -0.36208051 |
| LAX1 | 1.187541919 | 6.039223024 | 3.666236561 | 0.000631218 | 0.046394564 | -0.371406862 |
| DCDC2 | -1.483746024 | 4.797283316 | -3.646374599 | 0.000670258 | 0.0468019 | -0.424013472 |
| LYN | 1.044441179 | 9.310981096 | 3.634006735 | 0.000695728 | 0.047519582 | -0.456696591 |
| CXCL13 | 2.533683111 | 5.54655941 | 3.617615931 | 0.000730916 | 0.049074467 | -0.499921749 |
| RBP4 | -1.637519033 | 5.750629582 | -3.610641144 | 0.000746402 | 0.049283485 | -0.518284433 |
| FABP4 | -1.087215214 | 6.999695194 | -3.607541017 | 0.000753385 | 0.049524844 | -0.526440251 |
| DAPL1 | -1.733282623 | 6.433565615 | -3.600765615 | 0.000768867 | 0.049830946 | -0.544252188 |
| CYP4F8 | -1.084330699 | 6.167039358 | -3.59722725 | 0.000777072 | 0.049830946 | -0.55354724 |

**Table S2 The results of GO analysis in top 10 items.**

| ONTOLOGY | ID | Description | GeneRatio | BgRatio | pvalue | p.adjust | qvalue |
| --- | --- | --- | --- | --- | --- | --- | --- |
| BP | GO:0006882 | cellular zinc ion homeostasis | 6/151 | 39/18800 | 6.37E-07 | 0.000419336 | 0.000359189 |
| BP | GO:0030199 | collagen fibril organization | 7/151 | 62/18800 | 6.37E-07 | 0.000419336 | 0.000359189 |
| BP | GO:0045926 | negative regulation of growth | 12/151 | 245/18800 | 6.94E-07 | 0.000419336 | 0.000359189 |
| BP | GO:0055069 | zinc ion homeostasis | 6/151 | 41/18800 | 8.66E-07 | 0.000419336 | 0.000359189 |
| BP | GO:0030198 | extracellular matrix organization | 13/151 | 307/18800 | 1.22E-06 | 0.000419336 | 0.000359189 |
| BP | GO:0043062 | extracellular structure organization | 13/151 | 308/18800 | 1.26E-06 | 0.000419336 | 0.000359189 |
| BP | GO:0061448 | connective tissue development | 12/151 | 260/18800 | 1.30E-06 | 0.000419336 | 0.000359189 |
| BP | GO:0045229 | external encapsulating structure organization | 13/151 | 310/18800 | 1.36E-06 | 0.000419336 | 0.000359189 |
| BP | GO:0010043 | response to zinc ion | 6/151 | 53/18800 | 4.09E-06 | 0.001123848 | 0.000962649 |
| BP | GO:0010273 | detoxification of copper ion | 4/151 | 15/18800 | 5.10E-06 | 0.001146537 | 0.000982084 |
| CC | GO:0062023 | collagen-containing extracellular matrix | 26/153 | 429/19594 | 4.28E-16 | 8.70E-14 | 6.72E-14 |
| CC | GO:0005583 | fibrillar collagen trimer | 5/153 | 12/19594 | 2.06E-08 | 1.19E-06 | 9.20E-07 |
| CC | GO:0098643 | banded collagen fibril | 5/153 | 12/19594 | 2.06E-08 | 1.19E-06 | 9.20E-07 |
| CC | GO:0005581 | collagen trimer | 9/153 | 86/19594 | 2.35E-08 | 1.19E-06 | 9.20E-07 |
| CC | GO:0043292 | contractile fiber | 13/153 | 238/19594 | 4.81E-08 | 1.95E-06 | 1.51E-06 |
| CC | GO:0030016 | myofibril | 12/153 | 228/19594 | 2.41E-07 | 8.14E-06 | 6.29E-06 |
| CC | GO:0098644 | complex of collagen trimers | 5/153 | 22/19594 | 6.43E-07 | 1.87E-05 | 1.44E-05 |
| CC | GO:0030017 | sarcomere | 11/153 | 209/19594 | 7.77E-07 | 1.97E-05 | 1.52E-05 |
| CC | GO:0031674 | I band | 9/153 | 139/19594 | 1.47E-06 | 3.32E-05 | 2.57E-05 |
| CC | GO:0005884 | actin filament | 8/153 | 113/19594 | 2.94E-06 | 5.98E-05 | 4.62E-05 |
| MF | GO:0005201 | extracellular matrix structural constituent | 12/147 | 172/18410 | 1.35E-08 | 4.55E-06 | 3.85E-06 |
| MF | GO:0030020 | extracellular matrix structural constituent conferring tensile strength | 6/147 | 41/18410 | 8.35E-07 | 0.000140359 | 0.000118724 |
| MF | GO:0005504 | fatty acid binding | 5/147 | 49/18410 | 4.36E-05 | 0.004879422 | 0.004127331 |
| MF | GO:0048407 | platelet-derived growth factor binding | 3/147 | 11/18410 | 7.85E-05 | 0.006596154 | 0.005579454 |
| MF | GO:0061135 | endopeptidase regulator activity | 8/147 | 194/18410 | 0.000168249 | 0.010152943 | 0.008588015 |
| MF | GO:0005200 | structural constituent of cytoskeleton | 6/147 | 104/18410 | 0.000186663 | 0.010152943 | 0.008588015 |
| MF | GO:0036041 | long-chain fatty acid binding | 3/147 | 15/18410 | 0.00021152 | 0.010152943 | 0.008588015 |
| MF | GO:0033293 | monocarboxylic acid binding | 5/147 | 81/18410 | 0.000477065 | 0.017511137 | 0.014812052 |
| MF | GO:0061134 | peptidase regulator activity | 8/147 | 230/18410 | 0.000528581 | 0.017511137 | 0.014812052 |
| MF | GO:0005539 | glycosaminoglycan binding | 8/147 | 234/18410 | 0.000592059 | 0.017511137 | 0.014812052 |

**Table S3 The results of KEGG analysis.**

| ID | Description | GeneRatio | BgRatio | pvalue | p.adjust | qvalue |
| --- | --- | --- | --- | --- | --- | --- |
| hsa04657 | IL-17 signaling pathway | 6/79 | 94/8644 | 0.000206723 | 0.01831771 | 0.016761303 |
| hsa04974 | Protein digestion and absorption | 6/79 | 103/8644 | 0.000340163 | 0.01831771 | 0.016761303 |
| hsa04814 | Motor proteins | 8/79 | 193/8644 | 0.000359171 | 0.01831771 | 0.016761303 |
| hsa05410 | Hypertrophic cardiomyopathy | 5/79 | 97/8644 | 0.001877278 | 0.053418657 | 0.048879817 |
| hsa04978 | Mineral absorption | 4/79 | 60/8644 | 0.002138171 | 0.053418657 | 0.048879817 |
| hsa04933 | AGE-RAGE signaling pathway in diabetic complications | 5/79 | 100/8644 | 0.002146993 | 0.053418657 | 0.048879817 |
| hsa05414 | Dilated cardiomyopathy | 5/79 | 103/8644 | 0.002443991 | 0.053418657 | 0.048879817 |
| hsa04512 | ECM-receptor interaction | 4/79 | 89/8644 | 0.008773794 | 0.167798804 | 0.15354139 |
| hsa04510 | Focal adhesion | 6/79 | 203/8644 | 0.010454767 | 0.177731038 | 0.162629708 |
| hsa05146 | Amoebiasis | 4/79 | 102/8644 | 0.01397351 | 0.19592343 | 0.179276341 |
| hsa00670 | One carbon pool by folate | 2/79 | 20/8644 | 0.014085998 | 0.19592343 | 0.179276341 |
| hsa05132 | Salmonella infection | 6/79 | 249/8644 | 0.026002908 | 0.293559628 | 0.268616653 |
| hsa00591 | Linoleic acid metabolism | 2/79 | 30/8644 | 0.03041182 | 0.293559628 | 0.268616653 |
| hsa03320 | PPAR signaling pathway | 3/79 | 75/8644 | 0.031007159 | 0.293559628 | 0.268616653 |
| hsa05133 | Pertussis | 3/79 | 76/8644 | 0.032074317 | 0.293559628 | 0.268616653 |
| hsa04270 | Vascular smooth muscle contraction | 4/79 | 134/8644 | 0.033980122 | 0.293559628 | 0.268616653 |
| hsa05012 | Parkinson disease | 6/79 | 266/8644 | 0.034383531 | 0.293559628 | 0.268616653 |
| hsa05130 | Pathogenic Escherichia coli infection | 5/79 | 198/8644 | 0.034536427 | 0.293559628 | 0.268616653 |
| hsa04260 | Cardiac muscle contraction | 3/79 | 87/8644 | 0.045062803 | 0.354619415 | 0.324488354 |
| hsa04540 | Gap junction | 3/79 | 88/8644 | 0.046355479 | 0.354619415 | 0.324488354 |
| hsa04145 | Phagosome | 4/79 | 152/8644 | 0.050099491 | 0.357691863 | 0.327299744 |
| hsa05323 | Rheumatoid arthritis | 3/79 | 93/8644 | 0.053091723 | 0.357691863 | 0.327299744 |
| hsa05219 | Bladder cancer | 2/79 | 41/8644 | 0.053770672 | 0.357691863 | 0.327299744 |
| hsa04061 | Viral protein interaction with cytokine and cytokine receptor | 3/79 | 100/8644 | 0.063268451 | 0.403336374 | 0.369065964 |
| hsa05142 | Chagas disease | 3/79 | 102/8644 | 0.066331381 | 0.405948049 | 0.371455731 |
| hsa04350 | TGF-beta signaling pathway | 3/79 | 108/8644 | 0.075919917 | 0.431195892 | 0.394558333 |
| hsa05144 | Malaria | 2/79 | 50/8644 | 0.076255096 | 0.431195892 | 0.394558333 |
| hsa04913 | Ovarian steroidogenesis | 2/79 | 51/8644 | 0.078911667 | 0.431195892 | 0.394558333 |
| hsa00480 | Glutathione metabolism | 2/79 | 57/8644 | 0.095431654 | 0.503484242 | 0.460704535 |
| hsa05020 | Prion disease | 5/79 | 272/8644 | 0.102779794 | 0.524176947 | 0.479639036 |
| hsa00590 | Arachidonic acid metabolism | 2/79 | 61/8644 | 0.106946624 | 0.525373039 | 0.4807335 |
| hsa00140 | Steroid hormone biosynthesis | 2/79 | 62/8644 | 0.109881943 | 0.525373039 | 0.4807335 |
| hsa05205 | Proteoglycans in cancer | 4/79 | 205/8644 | 0.117626952 | 0.545361324 | 0.499023434 |

**Table S4 The results of DO analysis.**

| **ID** | **Description** | **GeneRatio** | **BgRatio** | **pvalue** | **p.adjust** | **qvalue** |
| --- | --- | --- | --- | --- | --- | --- |
| DOID:13359 | Ehlers-Danlos syndrome | 6/120 | 26/10312 | 4.17E-07 | 0.000156113 | 0.000120873 |
| DOID:0060084 | cell type benign neoplasm | 20/120 | 480/10312 | 5.94E-07 | 0.000156113 | 0.000120873 |
| DOID:3627 | aortic aneurysm | 10/120 | 113/10312 | 7.07E-07 | 0.000156113 | 0.000120873 |
| DOID:520 | aortic disease | 10/120 | 116/10312 | 9.02E-07 | 0.000156113 | 0.000120873 |
| DOID:854 | collagen disease | 7/120 | 57/10312 | 3.96E-06 | 0.000547942 | 0.000424251 |
| DOID:4450 | renal cell carcinoma | 18/120 | 472/10312 | 8.03E-06 | 0.00092667 | 0.000717486 |
| DOID:289 | endometriosis | 9/120 | 125/10312 | 1.43E-05 | 0.00141141 | 0.001092801 |
| DOID:0080307 | myofibrillar myopathy | 4/120 | 15/10312 | 2.16E-05 | 0.00186504 | 0.00144403 |
| DOID:13099 | Moyamoya disease | 4/120 | 17/10312 | 3.69E-05 | 0.002758187 | 0.00213556 |
| DOID:5041 | esophageal cancer | 12/120 | 255/10312 | 3.99E-05 | 0.002758187 | 0.00213556 |
| DOID:1107 | esophageal carcinoma | 11/120 | 219/10312 | 4.71E-05 | 0.002960292 | 0.002292042 |
| DOID:423 | myopathy | 17/120 | 498/10312 | 6.02E-05 | 0.003474109 | 0.002689871 |
| DOID:13580 | cholestasis | 9/120 | 161/10312 | 0.000104999 | 0.005589159 | 0.004327474 |
| DOID:11054 | urinary bladder cancer | 11/120 | 246/10312 | 0.000133625 | 0.006604884 | 0.005113912 |
| DOID:0090132 | complex cortical dysplasia with other brain malformations 7 | 3/120 | 10/10312 | 0.000173756 | 0.008015953 | 0.00620645 |
| DOID:1596 | mental depression | 8/120 | 138/10312 | 0.000200259 | 0.008196333 | 0.006346111 |
| DOID:13089 | intracranial arterial disease | 5/120 | 48/10312 | 0.000225324 | 0.008196333 | 0.006346111 |
| DOID:3527 | cerebral arterial disease | 5/120 | 48/10312 | 0.000225324 | 0.008196333 | 0.006346111 |
| DOID:0060260 | ptosis | 3/120 | 11/10312 | 0.000236888 | 0.008196333 | 0.006346111 |
| DOID:13934 | facial paralysis | 3/120 | 11/10312 | 0.000236888 | 0.008196333 | 0.006346111 |
| DOID:10286 | prostate carcinoma | 9/120 | 182/10312 | 0.00026516 | 0.008737665 | 0.006765244 |
| DOID:7693 | abdominal aortic aneurysm | 6/120 | 79/10312 | 0.000304688 | 0.009583836 | 0.007420403 |
| DOID:11335 | sarcoidosis | 7/120 | 116/10312 | 0.000396785 | 0.011938047 | 0.009243179 |
| DOID:4138 | bile duct disease | 9/120 | 195/10312 | 0.000440916 | 0.012248687 | 0.009483696 |
| DOID:9741 | biliary tract disease | 9/120 | 197/10312 | 0.000474966 | 0.012248687 | 0.009483696 |
| DOID:127 | leiomyoma | 7/120 | 120/10312 | 0.000487249 | 0.012248687 | 0.009483696 |
| DOID:0050997 | cerebellar ataxia, mental retardation and dysequlibrium syndrome | 3/120 | 14/10312 | 0.000509417 | 0.012248687 | 0.009483696 |
| DOID:1756 | facial nerve disease | 3/120 | 14/10312 | 0.000509417 | 0.012248687 | 0.009483696 |
| DOID:9835 | refractive error | 6/120 | 87/10312 | 0.000513312 | 0.012248687 | 0.009483696 |
| DOID:6713 | cerebrovascular disease | 12/120 | 337/10312 | 0.000541524 | 0.012272939 | 0.009502473 |
| DOID:14004 | thoracic aortic aneurysm | 4/120 | 33/10312 | 0.000549799 | 0.012272939 | 0.009502473 |
| DOID:11830 | myopia | 5/120 | 60/10312 | 0.000643177 | 0.013908703 | 0.010768984 |
| DOID:3070 | high grade glioma | 12/120 | 347/10312 | 0.000701581 | 0.014711945 | 0.011390904 |
| DOID:0080010 | bone structure disease | 4/120 | 36/10312 | 0.000770457 | 0.015550421 | 0.012040104 |
| DOID:2916 | hypersensitivity reaction type IV disease | 7/120 | 130/10312 | 0.00078651 | 0.015550421 | 0.012040104 |
| DOID:10952 | nephritis | 11/120 | 306/10312 | 0.000858662 | 0.016184266 | 0.012530866 |
| DOID:4007 | bladder carcinoma | 5/120 | 64/10312 | 0.000865344 | 0.016184266 | 0.012530866 |
| DOID:13832 | patent ductus arteriosus | 3/120 | 17/10312 | 0.000927707 | 0.016894041 | 0.013080418 |
| DOID:0090131 | complex cortical dysplasia with other brain malformations | 3/120 | 18/10312 | 0.00110384 | 0.019586081 | 0.015164763 |
| DOID:90 | degenerative disc disease | 3/120 | 19/10312 | 0.001299737 | 0.022065366 | 0.01708438 |
| DOID:1485 | cystic fibrosis | 8/120 | 183/10312 | 0.001307341 | 0.022065366 | 0.01708438 |
| DOID:3748 | esophagus squamous cell carcinoma | 7/120 | 143/10312 | 0.001374804 | 0.022651528 | 0.017538223 |
| DOID:0080007 | bone deterioration disease | 3/120 | 20/10312 | 0.001516191 | 0.024400101 | 0.018892077 |
| DOID:2994 | germ cell cancer | 13/120 | 439/10312 | 0.001745255 | 0.027448094 | 0.021252023 |
| DOID:0060056 | hypersensitivity reaction disease | 7/120 | 152/10312 | 0.00195203 | 0.030017887 | 0.023241717 |
| DOID:8398 | osteoarthritis | 9/120 | 243/10312 | 0.002084654 | 0.031360444 | 0.024281208 |
| DOID:3770 | pulmonary fibrosis | 8/120 | 198/10312 | 0.002151318 | 0.031674728 | 0.024524546 |
| DOID:9008 | psoriatic arthritis | 3/120 | 23/10312 | 0.002296318 | 0.033105248 | 0.025632143 |
| DOID:0050700 | cardiomyopathy | 13/120 | 454/10312 | 0.002346485 | 0.033138114 | 0.025657591 |
| DOID:3717 | gastric adenocarcinoma | 6/120 | 119/10312 | 0.002611023 | 0.03557189 | 0.027541971 |
| DOID:4766 | embryoma | 11/120 | 352/10312 | 0.002621628 | 0.03557189 | 0.027541971 |
| DOID:3082 | interstitial lung disease | 9/120 | 254/10312 | 0.002810514 | 0.037056385 | 0.02869136 |
| DOID:83 | cataract | 6/120 | 121/10312 | 0.002838134 | 0.037056385 | 0.02869136 |
| DOID:110 | lens disease | 6/120 | 123/10312 | 0.003079746 | 0.039208029 | 0.030357297 |
| DOID:1074 | kidney failure | 11/120 | 360/10312 | 0.003116245 | 0.039208029 | 0.030357297 |
| DOID:10534 | stomach cancer | 12/120 | 416/10312 | 0.003254737 | 0.040219252 | 0.031140248 |
| DOID:229 | female reproductive system disease | 12/120 | 421/10312 | 0.003584403 | 0.043056029 | 0.033336658 |
| DOID:11984 | hypertrophic cardiomyopathy | 6/120 | 127/10312 | 0.003608742 | 0.043056029 | 0.033336658 |
| DOID:0060036 | intrinsic cardiomyopathy | 11/120 | 371/10312 | 0.003916638 | 0.04517189 | 0.034974889 |
| DOID:0070004 | myeloid neoplasm | 11/120 | 371/10312 | 0.003916638 | 0.04517189 | 0.034974889 |
| DOID:799 | varicose veins | 3/120 | 28/10312 | 0.004071958 | 0.04619336 | 0.035765774 |
| DOID:688 | embryonal cancer | 11/120 | 378/10312 | 0.004506484 | 0.050090672 | 0.038783316 |
| DOID:3069 | malignant astrocytoma | 7/120 | 177/10312 | 0.004560278 | 0.050090672 | 0.038783316 |

**Table S5 The results of GSEA in IC/BPS dataset.**

| Description | setSize | enrichmentScore | NES | pvalue | p.adjust | qvalue |
| --- | --- | --- | --- | --- | --- | --- |
| KEGG_CELL_CYCLE | 114 | 0.670950924 | 2.036030724 | 2.06E-09 | 3.77E-07 | 2.80E-07 |
| KEGG_CYTOKINE_CYTOKINE_RECEPTOR_INTERACTION | 247 | 0.544127657 | 1.813468657 | 7.54E-08 | 6.90E-06 | 5.12E-06 |
| KEGG_OOCYTE_MEIOSIS | 106 | 0.638578494 | 1.924830913 | 1.87E-07 | 1.14E-05 | 8.47E-06 |
| KEGG_CHEMOKINE_SIGNALING_PATHWAY | 176 | 0.565637077 | 1.823317534 | 3.98E-07 | 1.82E-05 | 1.35E-05 |
| KEGG_COMPLEMENT_AND_COAGULATION_CASCADES | 68 | 0.696521237 | 1.97835884 | 6.28E-07 | 2.30E-05 | 1.70E-05 |
| KEGG_FOCAL_ADHESION | 188 | 0.550395329 | 1.785411045 | 1.19E-06 | 3.64E-05 | 2.70E-05 |
| KEGG_ECM_RECEPTOR_INTERACTION | 82 | 0.648112208 | 1.875310553 | 1.79E-06 | 4.67E-05 | 3.46E-05 |
| KEGG_DNA_REPLICATION | 33 | 0.781226857 | 1.981123987 | 5.83E-06 | 0.000133473 | 9.90E-05 |
| KEGG_PRION_DISEASES | 35 | 0.728705527 | 1.870511922 | 0.000104625 | 0.002127383 | 0.001578558 |
| KEGG_SYSTEMIC_LUPUS_ERYTHEMATOSUS | 53 | 0.651938978 | 1.798309734 | 0.000188531 | 0.00345012 | 0.002560055 |
| KEGG_DILATED_CARDIOMYOPATHY | 90 | 0.554314932 | 1.629174116 | 0.000302947 | 0.004619939 | 0.003428083 |
| KEGG_MAPK_SIGNALING_PATHWAY | 254 | 0.447463463 | 1.490336898 | 0.000300243 | 0.004619939 | 0.003428083 |
| KEGG_RIBOSOME | 78 | -0.557879979 | -1.745320228 | 0.000343377 | 0.004833687 | 0.003586687 |
| KEGG_CALCIUM_SIGNALING_PATHWAY | 171 | 0.487411 | 1.562145317 | 0.000383347 | 0.005010898 | 0.003718182 |
| KEGG_HEMATOPOIETIC_CELL_LINEAGE | 86 | 0.558355337 | 1.623179603 | 0.000674093 | 0.008223934 | 0.006102315 |
| KEGG_VASCULAR_SMOOTH_MUSCLE_CONTRACTION | 108 | 0.523544094 | 1.582753259 | 0.000733174 | 0.008385677 | 0.006222331 |
| KEGG_NEUROACTIVE_LIGAND_RECEPTOR_INTERACTION | 255 | 0.440994458 | 1.471493531 | 0.000847519 | 0.009123289 | 0.006769653 |
| KEGG_NOD_LIKE_RECEPTOR_SIGNALING_PATHWAY | 55 | 0.616841774 | 1.704622752 | 0.001061182 | 0.010788686 | 0.00800541 |
| KEGG_PATHOGENIC_ESCHERICHIA_COLI_INFECTION | 48 | 0.62782155 | 1.700494769 | 0.001230755 | 0.011854117 | 0.00879598 |
| KEGG_HYPERTROPHIC_CARDIOMYOPATHY_HCM | 83 | 0.558800338 | 1.617388987 | 0.001302674 | 0.011919471 | 0.008844474 |
| KEGG_TOLL_LIKE_RECEPTOR_SIGNALING_PATHWAY | 100 | 0.532888053 | 1.587597358 | 0.001403548 | 0.011956982 | 0.008872307 |
| KEGG_CELL_ADHESION_MOLECULES_CAMS | 129 | 0.500231602 | 1.5438754 | 0.001437451 | 0.011956982 | 0.008872307 |
| KEGG_GAP_JUNCTION | 80 | 0.550179096 | 1.587895853 | 0.002618365 | 0.020833076 | 0.015458538 |
| KEGG_LINOLEIC_ACID_METABOLISM | 27 | -0.696563049 | -1.781058976 | 0.002793857 | 0.021303157 | 0.015807347 |
| KEGG_PROTEASOME | 42 | 0.616388573 | 1.627481527 | 0.004077561 | 0.029847747 | 0.022147595 |
| KEGG_LEISHMANIA_INFECTION | 66 | 0.553862215 | 1.563200763 | 0.004372212 | 0.030773645 | 0.022834629 |
| KEGG_PROGESTERONE_MEDIATED_OOCYTE_MATURATION | 85 | 0.528169858 | 1.531201939 | 0.005563825 | 0.037710373 | 0.027981812 |
| KEGG_EPITHELIAL_CELL_SIGNALING_IN_HELICOBACTER_PYLORI_INFECTION | 65 | 0.565761886 | 1.590097799 | 0.006581642 | 0.043015732 | 0.03191849 |
| KEGG_ARRHYTHMOGENIC_RIGHT_VENTRICULAR_CARDIOMYOPATHY_ARVC | 74 | 0.530172508 | 1.513678917 | 0.007190538 | 0.045374773 | 0.033668943 |
| KEGG_ARACHIDONIC_ACID_METABOLISM | 53 | -0.53374499 | -1.545121797 | 0.008257842 | 0.048747905 | 0.036171871 |
| KEGG_LEUKOCYTE_TRANSENDOTHELIAL_MIGRATION | 108 | 0.474471336 | 1.434398865 | 0.008066708 | 0.048747905 | 0.036171871 |

**Table S6 There are significant genes in WGCNA analysis.**

| A1BG | ADH7 | LOC100130428 | CRTAM | ENC1 | SCGB1A1 | SELP | LAMP3 | CD101 | GLI1 | CLEC4E |
| --- | --- | --- | --- | --- | --- | --- | --- | --- | --- | --- |
| AADAT | ADRA1D | LOC100132686 | CSF1R | ENGASE | SCN4B | SEMA3C | LAP3 | CD151 | GPR162 | CLEC4F |
| AATK | ADRA2C | LOC157740 | CSF2RA | ENPP5 | SEMA3D | SEMA3E | LAPTM5 | CD177 | GREB1 | CLEC5A |
| ABCC3 | AFF3 | LOC389834 | CSF2RB | ENPP6 | SEMA6A | SEMA5A | LAT2 | CD24 | GREM2 | CLEC7A |
| ABCC4 | ALDH1A2 | LOC554207 | CSF3R | ENTPD3 | SGCA | SEMA7A | LAX1 | CD300LB | GRIK1-AS1 | CLECL1 |
| ABCC5 | ANLN | LOC613266 | CSGALNACT2 | ENTPD5 | SGMS2 | SERPINA5 | LCK | CD93 | GSTM5 | CLNK |
| ABCD3 | APOL4 | LOC646903 | CSMD3 | EPB41L4A-AS1 | SHCBP1 | SERPINB1 | LCP1 | CDA | GTSE1 | CMA1 |
| ABHD3 | ART5 | LOX | CST7 | EPB41L4B | SHISA3 | SERPINB11 | LCP2 | CDC42BPA | GUCA2B | CMAHP |
| ABRA | ASF1B | LOXL2 | CTLA4 | EPCAM | SIM1 | SERPINB5 | LEF1 | CDH1 | GULP1 | CMKLR1 |
| ACADL | ASPHD2 | LPAR3 | CTRL | EPHA1 | SKA1 | SERPINE1 | LGALS2 | CDH2 | HAPLN1 | CMTM2 |
| ACER1 | ASPM | LPIN1 | CTSC | EPHX1 | SKA3 | SERPINE2 | LILRA1 | CDH26 | HDAC10 | CNDP1 |
| ACOT11 | ATP12A | LPIN3 | CTSS | EPHX4 | SLC26A10 | SERPING1 | LILRA2 | CDK5R1 | HHIP | CNGA1 |
| ACOXL | ATP1B2 | LRMP | CTSW | EPM2AIP1 | SLC27A6 | SERTAD1 | LILRA4 | CDK5R2 | HHIPL1 | CNGA3 |
| ACSF2 | AURKA | LRP5 | CTSZ | ERAP1 | SLC2A4 | SFRP4 | LILRA5 | CDKN2A | HIST1H1B | CNR2 |
| ACSL5 | AURKB | LRRC15 | CX3CL1 | ERBB2 | SLC30A2 | SGCG | LILRB1 | CDON | HIST1H1E | CNTN1 |
| ACSM1 | AVPR2 | LRRC31 | CXCL10 | ERBB3 | SLC7A3 | SGIP1 | LILRB2 | CEACAM3 | HIST1H3I | CNTN2 |
| ACSM3 | AXIN2 | LRRC36 | CXCL11 | ERBB4 | SLCO1B3 | SGK2 | LILRB3 | CEBPB | HJURP | COL22A1 |
| ACTL9 | B3GAT1 | LRRC4 | CXCL13 | EREG | SLIT2 | SGPL1 | LILRB4 | CEBPE | HLF | COL4A4 |
| ACVR2A | BBOX1 | LRRD1 | CXCL9 | ERMP1 | SMPD3 | SGPP2 | LIMD2 | CELA1 | HMMR | CORO1A |
| ACVR2B | BIRC5 | LRRTM1 | CXCR1 | ERN2 | SNAP91 | SH2D1B | LIN7A | CEND1 | ID4 | COTL1 |
| ADAM22 | BRDT | LUZP2 | CXCR2 | ERP27 | SOD3 | SH2D4A | LIPC | CENPV | IGDCC4 | CP |
| ADAMTS14 | BRIP1 | LY6G6E | CXCR3 | ERRFI1 | SORL1 | SH3GL2 | LMNB1 | CEP19 | IRX3 | CPA3 |
| ADAMTS4 | BST1 | LY6H | CXCR4 | ESRRG | SP5 | SH3GLB2 | LOC100131496 | CEP290 | ITGA8 | CPNE5 |
| ADHFE1 | BTG2 | LYPD1 | CXCR5 | EYA1 | SPAG5 | SH3YL1 | LOC284379 | CFHR4 | ITGBL1 | CPVL |
| ADM | BUB1 | LYPD6 | CXCR6 | EYA4 | SPC24 | SHC4 | LOC399900 | CFTR | KANK4 | CR1 |
| ADRA1B | BUB1B | LYPD6B | CXorf21 | EZH2 | SPC25 | SHISA6 | LOC440934 | CGN | KBTBD11 | CR1L |
| AGBL2 | C16orf89 | LYVE1 | CXorf65 | F2RL2 | SPDEF | SHROOM1 | LOC441601 | CHI3L1 | KCNK2 | CR2 |
| AGR2 | C1QL1 | MACROD2 | CYBB | F3 | SPOCK1 | SIAH3 | LPCAT1 | CHP2 | KHDRBS2 | CRIP1 |
| AGR3 | C1QTNF4 | MADCAM1 | CYFIP2 | FAAH | SRPX | SIDT1 | LPXN | CHRDL2 | KIAA0101 | CRLF3 |
| AGT | C1QTNF7 | MAFF | CYP1A2 | FAM101B | SSTR1 | SIRPA | LRRC25 | CHRM3 | KIF11 | SPIB |
| AIRE | C4BPB | MAL2 | CYP4F8 | FAM149A | SYT1 | SIX1 | LRRC8E | CHRM4 | KIF14 | SPINK2 |
| AKAP1 | C7 | MAOA | CYSLTR1 | FAM163A | TAC1 | SIX4 | LRRK2 | CHST8 | KIF15 | SPINK4 |
| AKR1C1 | CA4 | MAP3K8 | CYTH4 | FAM167B | TAC3 | SLC10A2 | LST1 | CHST9 | KIF1A | SPNS3 |
| AKR1C3 | CADM2 | MARCO | CYTIP | FAM169A | TACR3 | SLC11A1 | LTA | CITED2 | KIF20A | SPOCK2 |
| ALCAM | CADM3 | MARVELD2 | DAZL | FAM174B | TBX2 | SLC13A3 | LTB | CKS2 | KIF2C | SRGN |
| ALDH1L1 | CASR | MAS1 | DDX60L | FAM189A1 | TCEAL2 | SLC14A1 | LTF | CLCF1 | KIF4A | SSH2 |
| ALDH3B2 | CBLN2 | MAST1 | DENND1C | FAM189A2 | TCF21 | SLC15A2 | LY86 | CLDN14 | KIF7 | ST8SIA4 |
| ALDH4A1 | CCK | MCCC1 | DENND3 | FAM20A | TCN1 | SLC16A10 | LY9 | CLDN3 | KIFC1 | STAP1 |
| ALDH5A1 | CCNA2 | MCTP1 | DENND4A | FAM3B | TDRD12 | SLC16A5 | LY96 | CLDN4 | KLF17 | STARD4 |
| ALDOB | CCNB1 | ME3 | DENND5A | FAM43B | TDRD9 | SLC16A6 | LYL1 | CLDN8 | KRT1 | STAT1 |
| ALOX5 | CCNB2 | MECOM | DERL3 | FAM71F2 | TFF1 | SLC16A9 | LYN | CLEC1A | KRT4 | STAT4 |
| ALOXE3 | CD1A | MEFV | DMXL2 | FAM83B | TFF2 | SLC19A2 | MAGIX | CLEC4G | LAMA5 | STEAP1 |
| ALPK2 | CD207 | MEIS2 | DNAJB9 | FAP | THBS2 | SLC22A18AS | MAK | CLIC2 | LCN2 | STK10 |
| ALPL | CD70 | METAP1 | DNAJC5B | FARP1 | TIMP2 | SLC22A4 | MAP4K1 | CLIC6 | LEFTY1 | STK17A |
| ALX3 | CDC20 | METTL7A | DNASE1L3 | FAT3 | TIMP3 | SLC22A5 | MAP4K2 | CLMN | LGI1 | STK17B |
| AMDHD1 | CDC25C | MEX3B | DOCK10 | FBP1 | TK1 | SLC25A10 | MAP7 | CMTM4 | LHX8 | STOX1 |
| AMIGO2 | CDC42EP2 | MFSD6L | DOCK11 | FBXL16 | TMEM119 | SLC25A27 | MB21D1 | CMYA5 | LINGO2 | STX11 |
| AMOT | CDC45 | MGC24103 | DOCK2 | FCAMR | TMEM130 | SLC27A2 | MCOLN2 | CNR1 | LOC100506990 | SULT1B1 |
| ANKFN1 | CDCA2 | MGST1 | DOCK3 | FCGBP | TMEM132C | SLC32A1 | MEF2C | CNTN3 | LRP8 | SUSD3 |
| ANKRD36 | CDCA3 | MIA2 | DOCK4 | FCN3 | TMOD1 | SLC38A4 | MEI1 | CNTNAP5 | LRRC18 | SUSD5 |
| ANKRD36B | CDCA5 | MID1 | DOCK5 | FER1L4 | TOP2A | SLC39A6 | MFNG | COL5A3 | LRRC38 | TACC3 |
| ANKRD45 | CDCA8 | MIPOL1 | DOK2 | FER1L6-AS1 | TOX3 | SLC44A3 | MGAM | COL9A3 | LRRC3B | TAGAP |
| ANKRD50 | CDH12 | MIR31HG | DOK3 | FERMT1 | TPH1 | SLC44A4 | MGC16025 | COMP | LRRC4B | TAP1 |
| ANKS4B | CDK1 | MLIP | DPEP2 | FGF10 | TPX2 | SLC46A2 | MICAL1 | CPA6 | LRRN4CL | TAP2 |
| ANO4 | CDKN1C | MLPH | DST | FGF11 | TRIP13 | SLC4A4 | MICB | CPAMD8 | MAD2L1 | TARP |
| ANO6 | CDKN3 | MMD | DTX1 | FGGY | TROAP | SLC5A7 | MLC1 | CPEB4 | MAGEL2 | TBC1D10C |
| ANXA10 | CDO1 | MMP1 | EAF2 | FLJ11710 | TTC25 | SLC6A3 | MLKL | CPNE4 | MAPK8IP1 | TBX21 |
| ANXA9 | CDT1 | MMP10 | EBI3 | FLJ34503 | TTK | SLC6A6 | MME | CPPED1 | MARC1 | TBXAS1 |
| AP1M2 | CEACAM6 | MMP12 | EBLN2 | FLJ37786 | TTR | SLC7A11 | MMP25 | CPXM1 | MB | TCF7 |
| AP1S3 | CENPE | MMP13 | EEF1A2 | FLNB | TYMS | SLC7A5 | MMP7 | CRABP2 | MCM10 | TCL1A |
| APLNR | CENPF | MMP3 | EFNA3 | FLRT3 | UBE2C | SLC7A8 | MMP9 | CREB3L3 | MELK | TCL1B |
| APOBEC3B | CENPK | MMRN1 | EFNB3 | FMO2 | UHRF1 | SLC9A2 | MNDA | CREB5 | MFAP4 | TDO2 |
| APOLD1 | CENPM | MOGAT3 | EGF | FMO3 | VIPR2 | SLC9A7 | MPEG1 | CREM | MGC10814 | TFAP2C |
| AQP3 | CENPW | MOP-1 | EHD1 | FMO5 | VSTM2A | SLCO4A1 | MPP1 | CRISPLD2 | MKI67 | TFCP2L1 |
| AR | CEP55 | MORC1 | ELMO1 | FMO9P | VSTM4 | SLITRK6 | MRC1 | CRLF1 | MND1 | TFEC |
| ARC | CFD | MPP7 | ELOVL2 | FNDC4 | VWDE | SMARCA1 | MS4A1 | CROT | MSLN | TGM2 |
| AREG | CHAC2 | MPPED2 | ELOVL5 | FOLR3 | WFDC1 | SMC4 | MS4A6A | CRYM | MUC13 | THEMIS |
| ARID5A | CILP2 | MPZL2 | EMB | FOS | WIF1 | SMR3A | MSN | CRYZ | MUCL1 | THRB |
| ARL14 | CKAP2 | MRAP2 | EMP2 | FOSB | WNT11 | SNAI1 | MT1M | CSF3 | MXRA5 | TIGIT |
| ARL5B | CKAP2L | MSC | EMP3 | FOSL1 | WNT9B | SNAP25 | MTHFD2 | CSRNP1 | MXRA8 | TIMD4 |
| ARMCX4 | CLDN11 | MSMB | ENTHD1 | FOXA1 | XRCC3 | SNCA | MTSS1L | CST2 | MYL10 | TLR1 |
| ARNTL2 | CLSPN | MSMO1 | ENTPD1 | FOXD1 | XRRA1 | SNCG | MUC16 | CTAG2 | MYL3 | TLR10 |
| ARSF | CNIH3 | MST1R | EOMES | FOXD3 | ZBTB16 | SNORA74A | MYCN | CTSE | MYOC | TLR2 |
| ASPN | COL12A1 | MSX2 | EPHB1 | FOXQ1 | ZDHHC8P1 | SNRK | MYO1F | CTXN1 | MYOZ3 | TLR4 |
| ASS1 | COL13A1 | MT1F | EPSTI1 | FOXR2 | ZFPM2 | SNX31 | MYO1G | CUX2 | NACAD | TLR6 |
| ASTN1 | COL24A1 | MT1G | ERAP2 | FREM2 | ZNF469 | SNX8 | MZB1 | CWH43 | NALCN | TLR7 |
| ATF3 | COL4A6 | MT1H | EVI2A | FRMPD4 | ZNF536 | SOCS1 | NAIP | CX3CR1 | NCAPG | ZNF267 |
| ATF7IP2 | COL6A1 | MT1X | EVI2B | FSIP1 | ZNF711 | SOCS3 | NAPSA | CXADR | NCAPH | ZNF280A |
| ATOH8 | COL6A2 | MT2A | F5 | FSIP2 | ZNF781 | SORD | NCF2 | CXCL1 | NDC80 | ZNF683 |
| ATP1A4 | COL6A5 | MTUS2 | FAM105A | FSTL4 | ZWINT | SOST | NCF4 | CXCL14 | NDNF | ZNF831 |
| ATP1B1 | COLEC12 | MUC1 | FAM129A | FUT7 | ABCC2 | SOSTDC1 | NCKAP1L | CXCL17 | NEFM | ZP1 |
| ATP2C2 | COLQ | MUC15 | FAM129C | FUT9 | ABCD2 | SOX2 | NCR3 | CXCL2 | NEK2 | ZFYVE9 |
| ATP7B | CORIN | MUC2 | FAM159A | FXYD3 | ACP5 | SOX3 | NEDD9 | CXCL3 | NOG | ZNF626 |
| ATP8B1 | CPN2 | MUM1L1 | FAM26F | FXYD4 | ACSL1 | SOX8 | NEURL3 | CXCL5 | NPFFR2 | ZNF662 |
| AVIL | CPNE7 | MX2 | FAM49A | FZD3 | ACTA1 | SPACA3 | NFAM1 | CXCL8 | NSAP11 | ZNF667 |
| AXDND1 | CRYGD | MXD1 | FAM65B | FZD5 | ADA | SPAG16 | NFE2 | CXorf57 | NTM | ZNF704 |
| B3GALT2 | CSGALNACT1 | MYADM | FAR2 | FZD6 | ADAM19 | SPATA17 | NFE4 | CYB5A | NTN5 | ZNF749 |
| B3GNT3 | CXCL12 | MYBL2 | FAS | G0S2 | ADAM20 | SPDYA | NFKBID | CYP1A1 | NTNG1 | ZNF780B |
| B3GNT5 | CYP2C18 | MYBPC1 | FASLG | GABRB3 | ADAM28 | SPECC1 | NFKBIE | CYP1B1 | NTRK2 | ZNF80 |
| BAMBI | CYP2D6 | MYBPC2 | FCAR | GAD2 | ADAM8 | SPHK1 | NKAIN2 | CYP24A1 | NTS | ZNF90 |
| BASP1 | CYS1 | MYBPH | FCER1G | GADD45B | ADAMDEC1 | SPINK1 | NKG7 | CYP2J2 | NUF2 | ZSCAN4 |
| BATF3 | DACH2 | MYEOV | FCER2 | GALNT12 | ADCY7 | SPINK6 | NLRC4 | CYP3A5 | NUSAP1 | ZSWIM4 |
| BCAS1 | DBH | MYO5C | FCGR2A | GALNT3 | AGAP2 | SPINT2 | NLRC5 | CYP4B1 | OIP5 | ABAT |
| BCAT2 | DCHS1 | N6AMT1 | FCGR2B | GALNT6 | AICDA | SPIRE2 | NLRP3 | CYP4F12 | OLFML3 | ADRB1 |
| BCKDHB | DEFA6 | NAALADL2 | FCHO1 | GAP43 | AIF1 | SPP1 | NLRP7 | CYP4F22 | OR8B8 | AMN1 |
| BDKRB1 | DEPDC1 | NAMPT | FCN1 | GARNL3 | AIFM3 | SPRNP1 | NMUR1 | CYP4V2 | ORC1 | ASCL2 |
| BDNF | DEPDC1B | NDST4 | FCRL1 | GATA2 | AIM2 | SPTLC3 | NOS2 | CYP4X1 | ORC6 | BCL2L15 |
| BEST2 | DIO3 | NEDD4L | FCRL2 | GATA3 | AKNA | SPTSSB | NPL | CYP4Z1 | OSR2 | BMP2 |
| BHLHE41 | DIO3OS | NEFL | FCRL3 | GATM | ALDH7A1 | SRD5A2 | NPTX1 | CYP4Z2P | PABPC5 | BPIFB1 |
| BHMT | DLGAP5 | NELL2 | FCRL4 | GCLC | ALOX15 | SRMS | NRG3 | DCDC2 | PAQR5 | BREA2 |
| BMPER | DMRTA1 | NEU4 | FCRL5 | GCM1 | ALOX5AP | SRPX2 | NTNG2 | DDAH1 | PBK | C16orf74 |
| BMX | DOK6 | NEUROG1 | FCRLA | GCNT4 | ALPK1 | SSH3 | NUP210 | DDIT4 | PCDHB9 | CA1 |
| BPGM | DPT | NFIL3 | FERMT3 | GDA | AMELY | SST | NXNL2 | DDX17 | PCP2 | CAPN12 |
| BPIFA1 | DRD1 | NFKB2 | FFAR2 | GDF15 | AMPD1 | ST3GAL5 | NYX | DDX3Y | PCSK2 | CCDC24 |
| BSPRY | DSEL | NFKBIZ | FGL2 | GDPD3 | ANGPTL6 | ST6GALNAC1 | OASL | DEFA5 | PDCD4 | CCNJL |
| BTBD16 | DTL | NGFR | FGR | GEM | ANGPTL7 | STAB2 | OGDHL | DEGS2 | PDE7B | CD1B |
| BTBD8 | E2F2 | NHSL2 | FKBP11 | GFRA3 | ANKRD18B | STAR | OLR1 | DEPTOR | PDGFRL | CDH18 |
| BTC | EBF2 | NINJ2 | FLI1 | GGT6 | ANKRD34A | STC1 | OR5P3 | DGKH | PDZD3 | CLDN16 |
| C10orf90 | EDIL3 | NLRP12 | FLT3 | GHSR | ANKRD44 | STEAP2 | ORM1 | DHCR24 | PEG10 | CLDN23 |
| C14orf28 | ELOVL7 | NME5 | FLVCR1-AS1 | GIPC2 | ANKRD55 | STEAP4 | OXGR1 | DHH | PGR | CLEC4GP1 |
| C14orf79 | ENPP4 | NNMT | FLYWCH2 | GJA5 | ANPEP | STK32A | P2RX1 | DHRS2 | PKMYT1 | COL9A2 |
| C16orf71 | EPDR1 | NOD2 | FMNL1 | GJB1 | ANXA6 | STMN2 | P2RX7 | DHRS3 | PLAC9 | SLC1A3 |
| C17orf105 | ESCO2 | NOV | FNBP1L | GJB3 | AOAH | STON2 | P2RY10 | DHRS9 | PLAT | SLC22A1 |
| C17orf96 | EVC | NOX1 | FOLR2 | GK3P | AP1S2 | STX19 | P2RY12 | DLGAP3 | PLIN4 | SLC24A4 |
| C17orf97 | EXO1 | NPHS1 | FOXP3 | GK5 | APBB1IP | SULF2 | P2RY13 | DLK1 | PLK1 | SLC25A37 |
| C1orf168 | F10 | NPHS2 | FPR1 | GLA | APOBR | SUSD4 | P2RY6 | DLL4 | PNCK | SLC26A4 |
| C1orf210 | FAM107A | NPTX2 | FPR2 | GLDN | APOC1 | SYDE2 | P2RY8 | DMBT1 | PNMA2 | SLC2A12 |
| C1orf220 | FAM111B | NR1H4 | FPR3 | GLT1D1 | APOE | SYNGR3 | PACSIN1 | DNAH5 | PNMT | SLC2A3 |
| C20orf96 | FAM150B | NR2F6 | FSD1 | GMNN | AQP9 | SYT13 | PADI2 | DOC2B | PODN | SLC35F3 |
| C2orf15 | FAM155B | NR4A2 | FXYD7 | GNG8 | ARHGAP24 | SYTL2 | PAG1 | DPEP1 | POU6F2 | SLC39A8 |
| C3orf14 | FAM181B | NR4A3 | FYB | GOLT1A | ARHGAP25 | SYTL5 | PARD3 | DPEP3 | PP12719 | SLC41A2 |
| C3orf36 | FAM19A2 | NRG1 | FYN | GPBAR1 | ARHGAP30 | TACSTD2 | PARP14 | DPH3P1 | PRC1 | SLC43A2 |
| C3orf52 | FAM19A5 | NRG4 | GAB3 | GPD1L | ARHGAP31 | TAS2R13 | PARP15 | DPP10 | PRDM6 | SLC6A12 |
| C3orf80 | FAM64A | NSUN6 | GAPT | GPHA2 | ARHGAP32 | TAS2R4 | PARP9 | DSC3 | PRPH | SLC7A7 |
| C7orf61 | FBLL1 | NTN4 | GBP2 | GPR160 | ARHGAP4 | TBX3 | PARVG | DSE | PRRT2 | SLCO2B1 |
| C8orf31 | FBLN1 | NUDT9P1 | GBP3 | GPR17 | ARHGAP9 | TCEA3 | PASK | DSG2 | PTN | SLCO4C1 |
| C9orf152 | FBLN2 | OCR1 | GBP4 | GPR182 | ARHGEF26 | TCTEX1D1 | PATL2 | DSP | PTPRD | SLCO5A1 |
| CAB39L | FBLN5 | ODAM | GBP5 | GPR37L1 | ARL11 | TDH | PAX5 | DTX4 | PTPRZ1 | SMAGP |
| CABP2 | FBLN7 | ODF3L1 | GCA | GPR39 | ARPC1B | TDRD5 | PCDH7 | DUSP1 | PTTG1 | SMAP2 |
| CALCRL | FBN2 | OGFRL1 | GFI1 | GPR4 | ARRB2 | TDRD6 | PDCD1 | DUSP10 | PTTG2 | SNAPC1 |
| CAPG | FBXL13 | OLFM4 | GFRA2 | GPRIN1 | ASAP1 | TEAD4 | PDCD1LG2 | DUSP5 | PTTG3P | SNCAIP |
| CAPN13 | FBXL21 | OLIG1 | GHRL | GPRIN2 | ASGR1 | TESC | PDE3B | DYSF | QPCT | SNX10 |
| CAPN9 | FBXO17 | OPALIN | GIMAP2 | GPX2 | ASGR2 | TFF3 | PDE4B | E2F8 | RAB3IL1 | SNX20 |
| CAPS | FGF9 | OR51E1 | GIMAP4 | GRHL2 | ASIP | TFPI2 | PDE6G | EDAR | RAD51 | SOAT2 |
| CAPS2 | FGFBP1 | OR5P2 | GIMAP7 | GRHL3 | ATG16L2 | TGFB1 | PDE7A | EDARADD | RAD51AP1 | SOD2 |
| CARD11 | FKBP10 | OR7A10 | GLIPR1 | GRIK1 | ATM | THBD | PDK1 | EDN1 | RAD54L | SP110 |
| CASQ1 | FNDC1 | OSBPL3 | GLIPR2 | GSDMB | ATP2A3 | THBS1 | PECAM1 | EFHC1 | RADIL | SPAG17 |
| CAT | FOXM1 | OSM | GLRX | GSTM1 | ATP8B4 | THSD4 | PER3 | EFNA5 | RBM24 | SPAG4 |
| CATSPER1 | FRRS1 | OSMR | GLTP | GSTM3 | ATP9A | TIGD3 | PERP | EGR1 | RBP4 | SPATA13 |
| CBLC | FRY | OSR1 | GMFG | GSTM4 | AZGP1 | TIGD4 | PHGDH | EGR2 | REN | SPI1 |
| CCDC42 | FXYD1 | OTOS | GNG2 | GUCY1B2 | BATF2 | TKTL1 | PHOSPHO1 | EGR3 | REPS2 | SLC1A3 |
| CCL1 | FZD7 | OVGP1 | GNGT2 | GZMH | BCL2A1 | TLE2 | PIK3AP1 | EGR4 | RGN | SLC22A1 |
| CCL17 | GABBR2 | OVOL2 | GNLY | GZMM | BEND4 | TM4SF1 | PIK3CD | EHF | RGS11 | SLC24A4 |
| CCL22 | GATA5 | P2RY14 | GPA33 | HAPLN3 | BEST1 | TM7SF2 | PIK3CG | EHHADH | RIBC2 | SLC25A37 |
| CCL24 | GATA6 | P4HA3 | GPAT2 | HAS1 | BEX5 | TMC4 | PIK3R5 | EID3 | RIPPLY2 | SLC26A4 |
| CCL25 | GATS | PABPC1L | GPNMB | HAS2 | BFSP2 | TMC7 | PILRA | ELF3 | RNF112 | SLC2A12 |
| CCL7 | GFRA1 | PACRG | GPR132 | HBEGF | BIN2 | TMED6 | PIM2 | ELF5 | RNF186 | SLC2A3 |
| CCL8 | GGH | PADI3 | GPR15 | HBM | BIRC3 | TMEM125 | PIP5K1B | ELK3 | RRM2 | SLC35F3 |
| CCNE2 | GINS2 | PALM3 | GPR155 | HEPACAM2 | BLK | TMEM132A | PITX1 | ELOVL6 | RYR3 | SLC39A8 |
| CCNO | GLDC | PAPSS2 | GPR171 | HES1 | BST2 | TMEM139 | PKHD1L1 | EMX2 | SCARA5 | SLC41A2 |
| PAQR4 | GPR174 | HGF | BTK | TMEM158 | PKIB | PLA2G2F | TLR8 | CRTAC1 | RPS6KA6 | SLC43A2 |
| PART1 | GPR18 | HHLA2 | BTLA | TMEM163 | PLA2G2D | PLCD3 | TLR9 | CTF1 | RSPO1 | SLC6A12 |
| PCBD1 | GPR183 | HIP1 | BTN2A2 | TMEM184A | PLA2G7 | PLIN1 | TM6SF1 | D2HGDH | RSPO3 | SLC7A7 |
| PCBP3 | GPR19 | HIST1H1C | BTN3A1 | TMEM190 | PLAC8 | PLK5 | TMC8 | DACT2 | RSPO4 | SLCO2B1 |
| PCDH17 | GPR65 | HIST1H1D | BTN3A2 | TMEM217 | PLB1 | PLXNB3 | TMEM140 | DISP2 | RTKN2 | SLCO4C1 |
| PCSK1 | GPR84 | HIST1H2AB | BTNL3 | TMEM232 | PLCB2 | PNPLA7 | TMEM150B | DLEC1 | S100A12 | SLCO5A1 |
| PDE10A | GPRIN3 | HIST1H2AM | C11orf53 | TMEM27 | PLCG2 | PPM1N | TMEM156 | DLX6 | S100A3 | SMAGP |
| PEX11A | GPSM3 | HIST1H2BC | C16orf54 | TMEM30B | PLCL1 | PPP1R32 | TMEM71 | DMBX1 | S100A8 | SMAP2 |
| PF4V1 | GRAMD1A | HIST1H2BD | C17orf99 | TMEM45B | PLCL2 | PROC | TMIGD2 | DUSP2 | S100A9 | SNAPC1 |
| PFKFB3 | GRAMD1B | HIST1H2BE | C1QA | TMEM54 | PLEK | PRSS30P | TNF | EXTL1 | S100B | SNCAIP |
| PFN2 | GRAP | HIST1H2BK | C1QB | TMEM59L | PLEKHO1 | PRSS50 | TNFAIP3 | FABP4 | S100P | SNX10 |
| PGAP1 | GRAP2 | HIST1H2BM | C1QC | TMEM63C | PLEKHO2 | RAB3B | TNFAIP8L2 | FABP6 | SAMD12 | SNX20 |
| PGLYRP1 | GRB14 | HIST1H2BO | C1S | TMEM79 | PLXNC1 | RASSF9 | TNFRSF13B | FGF20 | SAMD13 | SOAT2 |
| PHEX | GSTT1 | HIST1H3B | C1orf162 | TMEM88 | PMCH | RHPN1 | TNFRSF13C | FGFRL1 | SAMD9 | SOD2 |
| PHLDA1 | GTSF1 | HIST1H3F | C2 | TMEM97 | PNOC | RIMS2 | TNFRSF17 | FOXJ1 | SCCPDH | SP110 |
| PIGR | GUCA1C | HIST1H3G | C22orf34 | TMEM99 | POMC | ROBO1 | TNFRSF1B | GAD1 | SCG5 | SPAG17 |
| PIGZ | GUSBP11 | HIST1H4A | C2CD4A | TNFAIP2 | PPP1R3C | RPRM | TNFRSF4 | GAS2 | SCGB2A1 | SPAG4 |
| PITPNM3 | GVINP1 | HIST1H4H | C2CD4B | TNFAIP6 | PRAM1 | SEC11C | TNFRSF9 | GKN1 | SCIN | SPATA13 |
| PKHD1 | GZMA | HIST2H2BE | C3 | TNFRSF11B | PRDM8 | SHH | TNFSF11 | HES4 | SCMH1 | SPI1 |
| PKP2 | GZMB | HMCN1 | C3AR1 | TNFRSF12A | PREX1 | SHROOM3 | TNFSF13B | HOXB8 | SCN11A | ZNF626 |
| PLA2G2A | GZMK | HMGCS2 | C3orf62 | TNFRSF18 | PRF1 | SIX2 | TNFSF14 | HOXD1 | SCN2A | ZNF662 |
| PLA2R1 | HAVCR2 | HMOX1 | C3orf67 | TNFRSF21 | PRKCB | SLC1A4 | TNFSF8 | HOXD4 | SCRG1 | ZNF667 |
| PLAC4 | HCAR1 | HNF1B | C4BPA | TNFRSF8 | PRKCQ | SLC8A2 | TNIP3 | HS3ST1 | SCT | ZNF704 |
| PLAG1 | HCG27 | HNF4G | C5AR1 | TNFSF15 | PROK2 | SPOCD1 | TRAF1 | IGDCC3 | SCUBE2 | ZNF749 |
| PLAU | HCK | HNMT | C9orf72 | TNFSF9 | PRR5L | SPOCK3 | TRAF3IP3 | IGFBP2 | SDR42E1 | ZNF780B |
| PLAUR | HCLS1 | HOMER2 | CA12 | TNNC1 | PSMB9 | SPTBN5 | TRAM1L1 | IPO11 | SDS | ZNF80 |
| PLCE1 | HCP5 | HOOK2 | CA5A | TNRC18 | PSORS1C1 | SULT2A1 | TRANK1 | IRAK2 | SEC14L1 | ZNF90 |
| PLCH1 | HCST | HOXA11 | CALB1 | TOM1L1 | PSPH | SYT17 | TRAT1 | IRX1 | SELE | ZSCAN4 |
| PLEK2 | HELB | HOXA13 | CALHM3 | TP63 | PSTPIP1 | SYT8 | TREM1 | KBTBD12 | SELENBP1 | ZSWIM4 |
| PLEKHA6 | HERC5 | HOXA2 | CALML6 | TPPP3 | PSTPIP2 | TANC2 | TREML1 | KCNF1 | KCNA3 | ABAT |
| PLEKHG6 | HHEX | HOXA3 | CAMK2N1 | TRAK1 | PTCRA | TEKT5 | TREML2 | KCNH4 | KCNA5 | ADRB1 |
| PLEKHH1 | HILS1 | HOXA5 | CAMP | TREML4 | PTGER2 | TEPP | TRIM2 | KDELR3 | KCNAB2 | AMN1 |
| PLK3 | HIST1H1T | HOXA6 | CARD16 | TRIB1 | PTGER4 | TG | TRPM2 | KIFC2 | KCNJ10 | ASCL2 |
| PLLP | HK3 | HOXB13 | CASP1 | TRIM16 | PTGIR | TNNI2 | TRPV4 | KRTAP5-8 | KCNJ2 | BCL2L15 |
| PLOD2 | HLA-DMA | HOXB5 | CASP5 | TRIM17 | PTK2B | TNNT3 | TUBAL3 | KRTAP5-9 | KCNMA1 | BMP2 |
| PLS1 | HLA-DMB | HOXB6 | CASS4 | TRIM31 | PTPN21 | TNS4 | TXLNB | LOC100129112 | KCNS1 | BPIFB1 |
| PLXNB1 | HLA-DOA | HOXC9 | CC2D2B | TRIT1 | PTPN22 | TREH | TYROBP | MAPK8IP3 | KCTD1 | BREA2 |
| PM20D1 | HLA-DOB | HOXD10 | CCDC105 | TRPV6 | PTPN6 | TRNP1 | UBASH3A | MATN4 | KIAA0040 | C16orf74 |
| PMP2 | HLA-DPA1 | HOXD11 | CCL18 | TSGA10IP | PTPN7 | UCN | UCP2 | MEGF6 | KIAA0226L | CA1 |
| PNP | HLA-DPB1 | HOXD13 | CCL19 | TSPAN1 | PTPRC | WFDC13 | UGT8 | MPP6 | KIAA0895L | CAPN12 |
| POF1B | HLA-DPB2 | HP | CCL20 | TSPAN12 | PTPRCAP | WFIKKN1 | VAMP1 | MYH3 | KIAA0922 | CCDC24 |
| POU4F1 | HLA-DQA1 | HPGD | CCL21 | TSPAN15 | PTPRE | WNT8B | VAV1 | MYH7B | KIAA1217 | CCNJL |
| PPARG | HLA-DQB1 | HPR | CCL4 | TSPAN6 | PTPRJ | ZDHHC11 | VIM | MYO15A | KIAA1324 | CD1B |
| PPBP | HORMAD1 | HPSE | CCL5 | TSPAN8 | PTX3 | ZG16B | VNN1 | NOXA1 | KIF21B | CDH18 |
| PPFIBP2 | HRASLS2 | HRH2 | CCR1 | TSPYL5 | PVRIG | AADAC | VNN2 | NRG2 | KLF2 | CLDN16 |
| PPM1J | HSD11B1 | HRK | CCR10 | TTC39A | PYHIN1 | ABCA6 | VPREB3 | NSUN7 | KLHDC7B | CLDN23 |
| PPM1L | HSF5 | HS3ST5 | CCR2 | TTLL7 | RAB27A | ACCS | VSIG4 | PBX4 | KLHL13 | CLEC4GP1 |
| PPP1R15A | HSH2D | HS3ST6 | CCR3 | TUBA1A | RAB33A | ADAMTS19 | WARS | XCL1 | KLHL31 | COL9A2 |
| PPP1R1B | HTR1D | HS6ST2 | CCR4 | TUBB2B | RAB38 | ADAMTS8 | WAS | ZAP70 | KLHL6 | CLEC4E |
| PPP1R36 | HTRA4 | HSD11B2 | CCR6 | TUBB6 | RAB39B | ADAMTSL2 | WBSCR27 | ZBP1 | KLRB1 | CLEC4F |
| PPP1R9A | HVCN1 | HSPA6 | CCR7 | TULP1 | RAC2 | ADAMTSL3 | WDFY4 | ZBTB32 | KLRC3 | CLEC5A |
| PPP2R2B | ICAM1 | HTATSF1P2 | CCR8 | TXNRD1 | RAP1GAP | ADCY1 | WEE1 | ZC3H12D | KLRD1 | CLEC7A |
| PPP6R1 | ICAM3 | ICA1 | CD14 | UBE2DNL | RASAL3 | ADH1B | WIPF1 | ZEB2 | KLRF1 | CLECL1 |
| PRDM1 | ICAM4 | ICAM2 | CD163 | UBQLNL | RASGRP1 | RAD54B | IL2RA | ITLN1 | KLRG1 | CLNK |
| PRDM13 | ICOS | ICAM5 | CD180 | UBXN10 | RASGRP2 | RALGAPA2 | IL2RB | ITM2C | KMO | CMA1 |
| PRG2 | IDO1 | ID1 | CD19 | UCN2 | RASGRP3 | RANBP17 | IL2RG | ITPRIP | KRT20 | CMAHP |
| PRKAA2 | IDO2 | IDH1 | CD1C | UCP1 | RASSF2 | RASD1 | IL32 | JPH1 | LAG3 | CMKLR1 |
| PROK1 | IFI44L | IDI2-AS1 | CD1E | UPK1A | RASSF4 | RASEF | IL3RA | JSRP1 | LAIR1 | CMTM2 |
| PROM1 | IFI6 | IER3 | CD2 | UPK1B | RBP5 | RASGEF1B | IL5RA | JUN | KRT7 | CNDP1 |
| PRR15 | IFIH1 | IFI27 | CD22 | UPK2 | RCSD1 | RASGEF1C | IL6R | JUNB | KRT79 | CNGA1 |
| PRR15L | IFIT1 | IFNE | CD226 | UPK3A | RELB | RASGRP4 | IL7R | KANK3 | KRT8 | CNGA3 |
| PRSS12 | IFIT2 | IGFBP3 | CD244 | UPP1 | RELT | RASSF3 | INA | KCND2 | KRT81 | CNR2 |
| PRSS21 | IFIT3 | IGFBPL1 | CD247 | USP31 | REM2 | RASSF6 | INPP5D | KCNG1 | KRTAP11-1 | CNTN1 |
| PRSS33 | IFITM1 | IGFL2 | CD27 | USP35 | RENBP | RBM11 | INSM1 | KCNJ15 | KSR2 | CNTN2 |
| PRSS36 | IFITM2 | IGSF21 | CD274 | UST | RGL4 | RCAN1 | IQGAP2 | KCNJ3 | LAD1 | COL22A1 |
| PSCA | IFITM3 | IHH | CD28 | UTS2 | RGS1 | RCN1 | IRAK3 | KCNK12 | LAIR2 | COL4A4 |
| PSORS1C3 | IFNG | IKZF2 | CD300A | UTY | RGS17 | RDH10 | IRF1 | KCNK13 | LANCL3 | CORO1A |
| PTCH1 | IGFLR1 | IL10 | CD300C | VASN | RGS18 | REEP6 | IRF4 | KCNN4 | LCN15 | COTL1 |
| PTCHD1 | IGFN1 | IL11 | CD300LF | VAV3 | RHBDF2 | REG1A | IRF7 | KCNQ1 | LCN6 | CP |
| PTER | IGLL1 | IL13 | CD33 | VAX2 | RHOF | REG1B | IRF8 | KDM6B | LCTL | CPA3 |
| PTGDS | IGSF3 | IL13RA2 | CD36 | VCAN | RHOH | REG3A | ISG15 | KIAA1107 | LDLR | CPNE5 |
| PTGES | IGSF6 | IL17B | CD37 | VEPH1 | RILPL2 | RELN | ISG20 | KIAA1324L | LEAP2 | CPVL |
| PTGR1 | IGSF9 | IL17C | CD38 | VGF | RNASE2 | RETN | ISL2 | KIAA1456 | LGALS12 | CR1 |
| PTGS2 | IKZF1 | IL1B | CD3D | VGLL1 | RNASE3 | RGL3 | ITGA4 | KIAA1522 | LGALS4 | CR1L |
| PTP4A3 | IKZF3 | IL1R2 | CD3E | VILL | RNASE6 | RGMA | ITGAL | KIF23 | LGI2 | CR2 |
| PTPN13 | IL10RA | IL20RA | CD3G | VIP | RNF213 | RGS2 | ITGAM | KIF5C | LGR4 | CRIP1 |
| PTPRF | IL12RB1 | IL22RA2 | CD40LG | VIPR1 | RSAD2 | RGS9BP | ITGAX | KLF10 | LHFPL1 | CRLF3 |
| PTPRR | IL12RB2 | IL23A | CD48 | VMO1 | RTN1 | RHOBTB3 | ITGB2 | KLF5 | LHFPL5 | SEC31B |
| PTPRU | IL16 | IL24 | CD5 | VNN3 | RUNX3 | RHOV | ITGB7 | KLHDC7A | LIF | SECTM1 |
| PVALB | IL17F | IL6 | CD52 | VPS18 | S100Z | RIC3 | ITIH1 | KLHDC8A | LIFR | SELL |
| PYROXD2 | IL18BP | ILDR1 | CD53 | VSIG1 | S1PR4 | RNASE1 | ITK | KLHL5 | LILRA3 | SELPLG |
| QPRT | IL18RAP | INE1 | CD6 | VSIG2 | SAMD9L | RND1 | ITPR2 | KLK1 | LILRA6 | SEPT1 |
| RAB15 | IL21 | INSIG1 | CD69 | VSTM1 | SAMSN1 | RND3 | JAK3 | KREMEN2 | LIMCH1 | SEPT6 |
| RAB17 | IL21R | IRF6 | CD7 | VTCN1 | SASH3 | RNF128 | JAKMIP1 | KRT18 | LINC00174 | SERPINA1 |
| RAB25 | IL23R | ISL1 | CD72 | VWC2 | SBK1 | RNF43 | JUP | KRT19 | LIPG | SERPINB9 |
| RAB27B | IL26 | ITGA5 | CD79A | VWCE | SCD | ROPN1L | KANK1 | KRT32 | LIPH | SERPIND1 |
| RAB31 | IL27RA | ITGAD | CD79B | WDR63 | SCML4 | RPPH1 | KBTBD8 | KRT33B | LMX1A | SFMBT2 |
| CD80 | WDR72 | SGK1 | CIITA | ZNF302 | CECR1 | XIRP1 | SIGLEC7 | CHIT1 | ZNF204P | SLAMF6 |
| CD83 | WFDC10B | SGTB | CLC | ZNF320 | CECR5-AS1 | XK | SIGLEC9 | CHST11 | ZNF208 | SLAMF7 |
| CD84 | WFDC2 | SH2B3 | CLDND2 | ZNF334 | CECR6 | XYLB | SIRPB1 | CHST15 | ZNF214 | SLAMF8 |
| CD86 | WNT10B | SH2D1A | CLEC10A | ZNF404 | CELF2 | ZBED2 | SIRPB2 | CIDEA | ZNF295-AS1 | SLC15A3 |
| CD8A | WNT16 | SH2D2A | CLEC12A | ZNF486 | CES4A | ZBTB7C | SIRPD | CEACAM4 | WWC1 | SIGLEC5 |
| CD9 | WNT2 | SH2D3C | CLEC12B | ZNF507 | CETP | ZDHHC2 | SIRPG | CLEC4D | ZNF610 | CHI3L2 |
| CD96 | WNT5A | SH3BGRL2 | CLEC2B | ZNF514 | CFB | ZFAND2A | SIT1 | SLAMF1 | ZNF154 | CEACAM21 |
| CDC42EP3 | WNT7A | SIGLEC1 | CLEC4A | ZNF540 | CFP | ZFP14 | SLA | WNT7B | SIGLEC10 | CLEC4C |
| ZNF552 | CHGB | ZFP36 | SLA2 |  |  |  |  |  |  |  |

**Table S7 The overlapping genes from these three machine learning algorithms.**

| **SVM-RFE Gene** | **RF Gene** | **LASSO Gene** | **InterGenes** |
| --- | --- | --- | --- |
| PAX5 | PAX5 | PLAC8 | PLAC8 |
| KCNJ15 | ABCC4 | S100A8 | S100A8 |
| HMGCS2 | PLAC8 | KIF23 | PPBP |
| CEACAM6 | S100A8 | KCNJ15 |  |
| PLAC8 | SORL1 | CRISP3 |  |
| ABCC4 | FAM19A2 | PPBP |  |
| SIX2 | HMGCS2 | FABP4 |  |
| SLITRK6 | SERPINA1 |  |  |
| FAM19A2 | UST |  |  |
| FPR1 | HMMR |  |  |
| AQP9 | CEACAM6 |  |  |
| SERPINA1 | PPBP |  |  |
| NIPAL4 |  |  |  |
| PPBP |  |  |  |
| PBK |  |  |  |
| S100A8 |  |  |  |
